# Maintaining a wild phenotype in a conservation hatchery program for Chinook salmon: The effect of managed breeding on early male maturation

**DOI:** 10.1371/journal.pone.0216168

**Published:** 2019-05-15

**Authors:** Donald A. Larsen, Deborah L. Harstad, Abby E. Fuhrman, Curtis M. Knudsen, Steven L. Schroder, William J. Bosch, Peter F. Galbreath, David E. Fast, Brian R. Beckman

**Affiliations:** 1 Environmental and Fisheries Sciences Division, Northwest Fisheries Science Center, National Marine Fisheries Service, National Oceanic and Atmospheric Administration, Seattle, Washington, United States of America; 2 Oncorh Consulting, Olympia, Washington, United States of America; 3 Washington Department of Fish and Wildlife, Olympia, Washington, United States of America; 4 Yakama Nation Fisheries, Toppenish, Washington, United States of America; 5 Columbia River Inter-Tribal Fish Commission, Portland, Oregon, United States of America; Universitat de Barcelona, SPAIN

## Abstract

In many salmonid species, age and size at maturation is plastic and influenced by the interaction between genetic and environmental factors. Hatchery reared salmon often mature at an earlier age and smaller size than wild fish. Modern salmon conservation efforts have focused on managing the level of gene flow between hatchery and natural origin fish to minimize potential genotypic and phenotypic change. In salmonids, maturation probability is dependent on exceeding a genetically set threshold in growth rate and energetic status (and by association, body size) referred to as the probabalisitic maturation reaction norm (PMRN). Over fourteen years, we monitored the frequency of age-2 precocious male maturation (common term: age-2 minijack rate) and the PMRN of natural founder (FNDR), integrated natural-hatchery (INT), and segregated hatchery (SEG) broodlines of spring Chinook salmon, *Oncorhynchus tshawytscha*. The average age-2 minijack rate (± SEM) of the FNDR, INT and SEG broodlines was 48.2 ± 5.2%, 41.9 ± 3.6% and 30.9 ± 4.7%, respectively. Additionally, the PMRN W_*P*50_ (predicted weight at 50% maturation) of the SEG broodline was significantly greater (20.5 g) than that of the FNDR/INT broodlines (18.2 g). We also conducted a common garden experiment exploring the effects of less than one [INT (0–1)], one [SEG (1)] or two [SEG (2)] generations of hatchery culture on the age-2 minijack rate and PMRN W_*P*50_. Growth was not significantly different among broodlines, but age-2 minijack rates were significantly lower following two consecutive generations of hatchery culture: [INT (0–1): 68.3 ± 1.7%], [SEG (1): 70.3 ± 1.8%] and [SEG (2): 58.6 ± 0.4%] and the PMRN W_*P*50_ was significantly higher by 6.1 g after two generations of SEG culture. These results indicate that managed gene flow reduces phenotypic divergence, but may serve to maintain potentially undesirably high age-2 minijack rates in salmon conservation hatchery programs.

## Introduction

Declines in salmon abundance throughout the United States, coupled with the regulatory requirements of the Endangered Species Act (Federal Register 70:37160), have resulted in efforts to enhance and restore threatened and endangered salmon populations. Salmon hatcheries have been in common use for the past century as a familiar response to the threat of declining stocks [1]. While these programs may produce significant quantities of returning adults, various negative effects associated with hatchery culture of salmonids have been documented [2]. One major concern is evidence demonstrating reduced reproductive fitness of hatchery fish when spawning in the natural environment [3, 4, 5, 6] with the fitness loss occurring in as little as one generation in culture [7, 8]. Domestication selection, genetic drift, and inbreeding depression are possible mechanisms responsible for this fitness loss [9, 10, 11, 12].

New approaches to the captive culture of salmon have been developed in an attempt to minimize differences between hatchery and wild fish, the most common of which is the integrated hatchery program. In an integrated program, gene flow between hatchery and wild fish is managed such that either a portion or all of the broodstock are sourced from unmarked returning adults that were born and reared in the wild, and returning hatchery adults are allowed to spawn naturally in the river. Fundamentally, a supplementation program is designed to provide the advantage of increased egg-to-juvenile survival typical of standard hatchery rearing while presumably minimizing domestication selection [13]. Supplementation hatcheries have been embraced by agencies responsible for enhancing salmon populations for both endangered and unlisted stocks [14]. However, the ability of these programs to produce fish without altering their essential wild-like genotype and phenotype (avoiding domestication) is only recently being elucidated [15, 16, 17, 18, 19].

The age at which an animal undergoes puberty is one of the most important components of its ontogeny and salmonid species that express complex life histories are no exception. Exposure to hatchery culture conditions, including optimal water temperatures and high feeding rates with concomitant high growth rates, often result in earlier age at sexual maturity when compared with their naturally rearing conspecifics, particularly for males [20]. In spring (or stream-type) Chinook salmon, *Oncorhynchus tshawytscha*, [21] male maturation can occur at age-1 (common names: precocious parr, microjack), age 2 (minijack), age-3 (jack), age 4 or 5 years post fertilization (see Larsen et al. [22] for definitions). Males that mature “precociously” as age-1 microjacks or age-2 minijacks are two or more orders of magnitude smaller in weight than anadromous (ocean rearing) adults. While the presence of these alternate male life history types is considered relatively rare in naturally rearing populations of spring Chinook salmon [22, 23, 24, 25], their presence among hatchery and experimental groups can be very high, reaching levels in excess of 90% of all males [22, 26, 27, 28, 29]. Recent studies employing a range of methodologies including population modeling [30], pedigree analysis [6], and Columbia River basin hatchery surveys [29] have all suggested that early male maturity may be an important contributor to domestication selection in hatchery Chinook salmon.

Age at maturity in salmonids is controlled by both genetic and environmental factors [31, 32, 33]. The size and age at which salmonids make the physiological committment to initiate maturation has been described as a conditional strategy in which variation in life history type is under polygenic control and expression of a phenotype depends on exceeding some threshold condition (reviewed in Hutchings [34] and Dodson et al. [35]). Studies have shown that individuals whose size, growth rate and energy stores exceed this genetically determined threshold at a specific age are likely to initiate the maturation process [32, 36, 37, 38]. This relationship between body size and the probability of maturing at a given age is often referred to as the probabalistic maturation reaction norm (PMRN; [39]). The age-specific body size at which the probability of maturing is 50% (body weight at 50% probability of maturation) has been used in numerous studies as comparative evidence of evolutionary change in the PMRN [35]; hereafter, PMRN W _*P*50_.

In this study we wanted to answer the following question: Can newly implemented integrated hatchery programs avoid changes in age of maturation in the populations being conserved? We hypothesized that under traditional segregated hatchery protocols, which do not incorporate age-1 microjacks or age-2 minijacks into the broodstock, there may be strong selection acting to reduce the incidence of the age-2 minijack phenotype and, in effect, increase the theshold size (PMRN W _*P*50_) neccessary for initiating age-2 minijack maturation. In contrast, while integrated hatchery protocols don’t incorporate age-1 micojacks or age-2 minijacks in the boodstock in the hatchery either, the opportunity for genetic contribution from these life-history types when allowed to spawn in the wild exists. Thus, we examined the initiation and development of an integrated hatchery program for spring Chinook salmon in the Columbia River basin. We assessed progeny from the original founding broodstock [Founders (FNDR)) and two succeeding generations (Integrated (INT)]. In addition, we monitored progeny from a small experimental group that was generated using a typical segregated breeding design (SEG). We had the following expectations: (1) we would detect domestication effects on early male maturation in progeny from the SEG broodline relative to the FNDR and INT broodlines; and (2) we would detect little or no evidence of domestication effects on early male maturation between progeny of the FNDR and INT broodlines.

Our analytical approach was based on the premise that age of maturation is a phenotype determined by genetic × environmental interactions. To accurately assess for potential differences in phenotypes across years and generations we had to account for both genetic and environmental factors. The genetic factors included: (1) Hatchery broodline (FNDR, INT, SEG). (2) Proportion age-3 jacks in the broodstock. Previous studies have demonstrated that fertilization of eggs with milt from age-3 jack males increases the probability of their male progeny maturing as age-3 jacks [40], but the extent to which age-3 jack contribution affects age-2 minijack rates in progeny is currently unknown. (3) Brood year [a majority of anadromous natural origin fish within a cohort will return to the Yakima River, WA (where the study was conducted) within the same year (average return rate at age 4 is 87%; [17])]. The environmental factors included water temperature and feed ration which directly impact variation in fish size and seasonal growth rates across brood years.

Two approaches were implemented in this study: In Part I, we tested whether the age-2 minijack rate decreased, and the PMRN W_*P*50_ increased, in the SEG broodline consistent with domestication at the hatchery and assessed whether the aformentioned genetic and environmental factors may influence them. In Part II, we conducted a controlled, common garden, rearing experiment to test whether progeny of the SEG broodline have decreased age-2 minijack rates and an increased PMRN W_*P*50_ consistent with domestication after one or two generations in culture.

## Materials and methods

### Ethics statement

Part I of this investigation, was conducted under permission and cooperation of resource co-managers of the Yakama Nation Fisheries and the Washington Department of Fish and Wildlife. Part II, was conducted at the Northwest Fisheries Science Center (NWFSC), Seattle in accordance with University of Washington Institutional Animal Care and Use Committee (IACUC) protocol number 2313–90. This study did not involve any threatened, endangered or protected species.

### Study site

The Cle Elum Supplementation and Research Facility (CESRF) was designed and implemented in 1997 to test the efficacy of using supplementation to rebuild the depressed natural spring Chinook salmon population in the upper Yakima River of Washington State, USA while minimizing genetic and ecological risks associated with captive culture ([17]; Table 1). The program uses only natural origin adults for broodstock each year, initially creating a founding (FNDR) broodline in the first seven years [brood years (BYs) 1997–2003] and then an integrated (INT) hatchery broodline from BYs 2004–2011. [Although the INT broodline uses unmarked (natural-origin) individuals for broodstock, it could potentially have genetic influence from hatchery-origin individuals that returned and spawned in the Yakima River in previous generation(s)]. Hatchery offspring from the FNDR/INT broodlines are adipose fin clipped and tagged [coded-wire-tags, passive integrated transponder (PIT tags, Biomark, Boise, ID) and colored visible implant fluorescent elastomer eye tags (Northwest Marine Technologies, Inc., Shaw Island, WA)] prior to release from the hatchery and when they return to the river as adults they are not brought back into the hatchery, but rather allowed to spawn naturally in the river. The percentage of natural spawners that are of hatchery origin has ranged from 20% to 76% (mean = 56%) from 2001 to 2013 [17]. Starting in 2002 the CESRF also established an experimental segregated (SEG) broodline from first generation returning hatchery adults. Offspring from the SEG broodline are also reared to the smolt stage and uniquely marked and tagged such that they can all be collected prior to spawning and artifically spawned in the hatchery with only SEG broodline fish (Table 1). Thus, the CESRF provided a unique opportunity to explore how effective managed gene flow (integration vs. segregation) may be at minimizing both phenotypic [17] and genotypic [16] differences associated with hatchery culture.

**Table 1:** Chronology of development of hatchery ancestry for Yakima River spring Chinook salmon through the first four generations of Cle Elum Supplementation and Research Facility (CESRF) operation.

Return Year	First generation^a^				Second generation^b^				Third generation^c^				Fourth generation			
	Brood Year:															
	1997	1998	1999	2000^d^	2001	2002^e^	2003	2004^f^	2005	2006	2007^g^	2008	2009	2010	2011	2012
2000	3															
2001	4	3														
2002	5	4	3													
2003		5	4	3												
2004			5	4	3											
2005				5	4	3										
2006					5	4	3									
2007	** **					5	4	3								
2008							5	4	3							
2009								5	4	3						
2010									5	4	3					
2011										5	4	3				
2012											5	4	3			
2013												5	4	3		
2014													5	4	3	
2015														5	4	3
2016															5	4
2017																5

Entries denote age at return for each brood year. Gray bars indicate which brood classes were used for Part I comparison. The black box represents the adult returns used as broodstock for Part II, the common garden experiment. Generation time assumes that most adults return at age 4 to spawn (see Fast et al. [17]).

^a^ Initiation of CESRF operation and broodstock collection began in 1997.

^b^ Hatchery-reared fish began returning to spawn in the Yakima River.

^c^ First returns of natural-origin fish produced by naturally-spawning hatchery fish.

^d^ Some small contribution from age-3 hatchery males (jacks) spawning in the Yakima River in 2000 was possible.

^e^ The SEG broodline was established from first generation returning hatchery adults in 2002.

^f^ The transition from FNDR to INT broodlines is conservatively set at BY 2004, although the chance of hatchery-influence from an age-3 jack grandparent or parent is still relatively low.

^g^ The common garden experiment (Part II) was conducted on INT (0–1), SEG (1), and SEG (2) progeny from brood that returned in 2007.

### Adult broodstock and juvenile fish rearing

**Fig 1:**
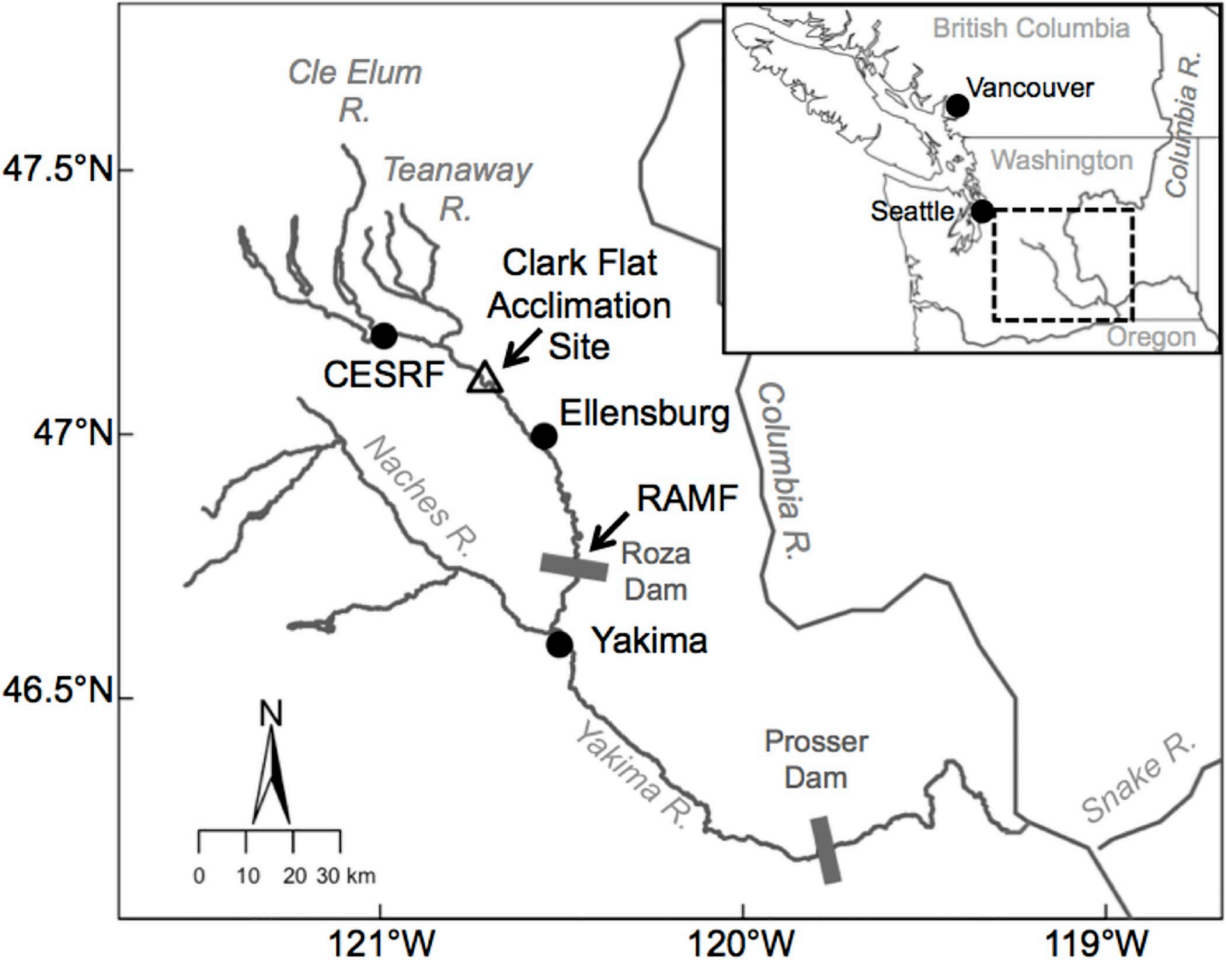
Location of study sites in the Yakima River basin (dashed box in inset), WA, USA.

Adult spring Chinook salmon were collected annually at the Roza Dam Adult Monitoring Facility (RAMF) on the Yakima River [river kilometer (rkm) 208, measured from the confluence with the Columbia River] between April and September (Fig 1; Table 1). The FNDR and INT broodline were sourced from untagged fish (adipose fin present) that were born and reared in the river and randomly selected proportionally throughout the adult return period. The SEG broodline was sourced from uniquely marked (adipose fin absent) and tagged (coded-wire-tags, PIT tags) and colored visible implant fluorescent elastomer eye tags as described above. At the Cle Elum Hatchery (rkm 297), progeny from the FNDR or INT broodlines were reared in 16 hatchery raceways (~45,000 fish/raceway = 720,000 fish total). Progeny from the SEG broodline were reared in two raceways (~45,000 fish/ raceway = 90,000 fish total). For a full description of the broodstock collection and spawning protocols see Knudsen et al. [41, 42] and for juvenile rearing and acclimation see Larsen et al. [28, 43] and Fast et al. [44].

Returning adult spring Chinook salmon are collected at the Roza Adult Monitoring Facility (RAMF) adjacent to Roza Dam on the Yakima River. Juveniles are reared at the Cle Elum Supplementation and Research Facility (CESRF) near Cle Elum, WA. Fish used in this study were transferred to the Clark Flat acclimation site prior to volitional release.

After approximately one year of rearing at the CESRF, all progeny were moved to one of three remote acclimation sites (Clark Flat, Easton, Jack Creek) that are distributed throughout the upper Yakima River basin and equipped with six raceways each. The Easton and Jack Creek sites were populated with only FNDR (BYs 1997–2003) and INT (BYs 2004–2011) fish. The two SEG (BYs 2002–2011) raceways were always transferred to the Clark Flat acclimation site (rkm 272) along with four raceways containing FNDR (BYs 1997–2003) and INT (BYs 2004–2011) fish. In early March of each year, after approximately six weeks of rearing at the acclimation sites, all fish were permitted to volitionally emigrate from the facilities until the end of May, after which all remaining fish were then flushed into the river.

### Part I: Comparing age-2 minijack rates and PMRN of Yakima River FNDR, INT and SEG progeny at Clark Flat acclimation site

**Fig 2:**
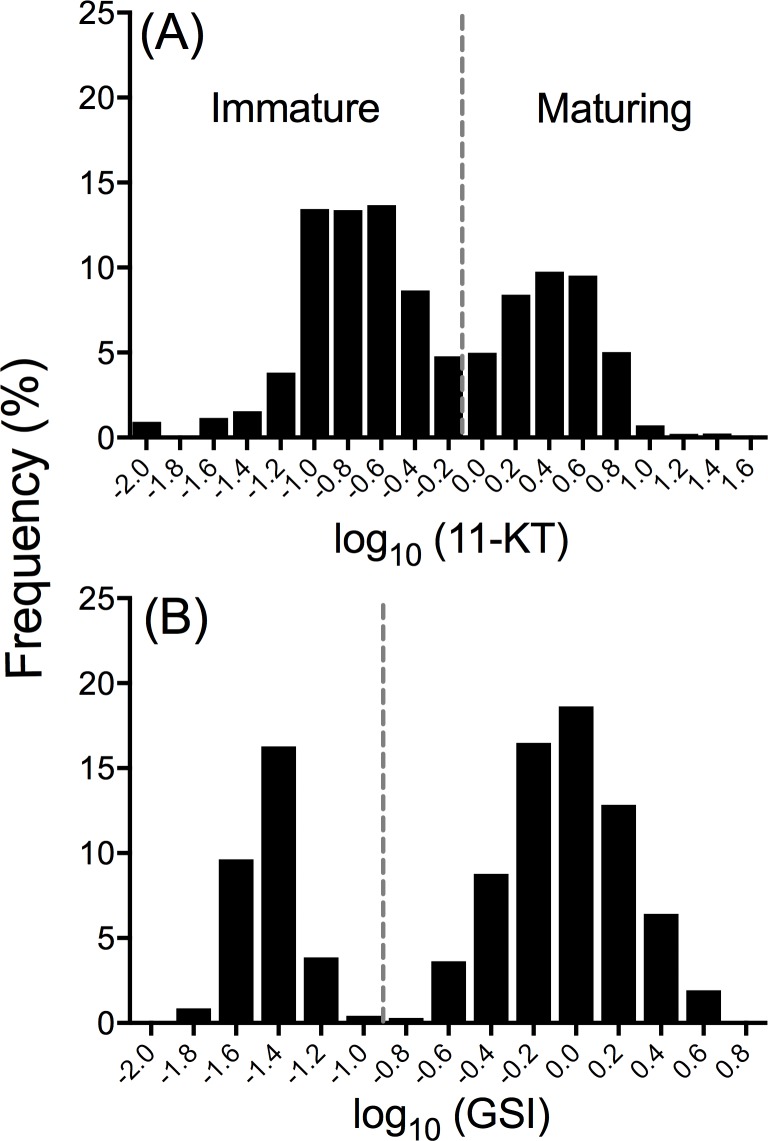
11-KT and GSI frequency distributions of immature and maturing yearling Yakima River spring Chinook salmon males. **(A)** log_10_ 11-KT values for male fish reared at Clark Flat acclimation site across BYs 1998–2011 (Part I); and **(B)** log_10_ GSI values for male fish from the common garden experiment (Part II). Dashed lines represent the cut off value used to identify immature males from those maturing at age 2.

The number of fish sampled from the FNDR, INT and SEG broodlines varied according to brood year (Table 2). Age-2 minijack rates were estimated in progeny from the FNDR broodline in BYs 1998–2003, INT broodline in BYs 2004–2011, and SEG broodline in BYs 2002–2011 (Table 2). The FNDR broodline was established in 1997, but age-2 minijack rates in that year were not determined. The age-2 minijack rates were determined at the Clark Flat acclimation site at approximately 18 months post spawning, just prior to release in early March of years 2000–2013. Fish were randomly netted from the raceways and individually euthanized using a buffered solution of 0.05% MS-222 (tricaine methanesulfonate; Argent Chemical Laboratories, Redmond, WA) to alleviate fish stress and suffering. They were measured for fork length in millimeters (mm) and body weight in grams (g). Blood was collected from the caudal vein using heparinized Natelson tubes (VWR, Radnor, PA) after severing the caudal peduncle. Blood was centrifuged for 5 min at 3,000 × g to isolate plasma. Plasma was kept frozen at -80°C until analysis. Gonads were visually inspected for determination of sex as described in Larsen et al. [28]. Final determination of maturation status was completed by measuring plasma levels of the sex steroid 11-ketotestosterone (11-KT) using an enzyme-linked immunosorbent assay according to the method of Cuisset et al. [45]. Males with 11-KT levels exceeding the predetermined threshold of 0.8 ng/ml were considered to be maturing as age-2 minijacks as described by Larsen et al. [22, 28] (Fig 2A).

**Table 2:** Population summary (sex counts, mean size) from age-2 minijack assessments at Clark Flat acclimation site.

Brood Year	Sample Date	Brood-line	M	MD	F	Total	%MD	Mean Length (mm)	SE, Length	Mean Weight (g)	SE, Weight
1998	3/14/00	FNDR	9	14	9	32	60.9	115.3	1.43	17.8	0.76
1999	3/13/01	FNDR	100	67	193	360	40.1	117.9	0.47	18.3	0.23
2000	3/19/02	FNDR	108	78	174	360	41.9	122.0	0.50	20.8	0.28
2001	3/12/03	FNDR	21	35	64	120	62.5	118.8	0.79	19.0	0.42
2002	3/10/04	SEG	24	4	32	60	14.3	113.6	0.82	15.3	0.37
		FNDR	25	29	66	120	53.7	115.3	0.75	16.0	0.33
2003	3/7/05	SEG	35	6	19	60	14.6	114.1	0.82	15.8	0.38
		FNDR	44	19	57	120	30.2	111.9	0.77	15.0	0.31
2004	3/8/06	SEG	46	9	65	120	16.4	111.9	0.65	15.3	0.28
		INT	34	27	59	120	44.3	112.6	0.64	15.3	0.29
2005	3/5/07	SEG	99	28	113	240	22.0	112.2	0.56	16.0	0.28
		INT	98	33	109	240	25.2	110.5	0.48	14.8	0.22
2006	3/4/08	SEG	55	65	120	240	54.2	114.5	0.57	15.8	0.25
		INT	79	52	109	240	39.7	113.1	0.58	15.3	0.26
2007	3/9/09	SEG	100	32	108	240	24.2	115.8	0.51	16.4	0.24
		INT	76	55	109	240	42.0	118.2	0.51	18.0	0.26
2008	3/8/10	SEG	95	42	103	240	30.7	117.9	0.47	17.8	0.24
		INT	90	42	108	240	31.8	116.0	0.50	17.0	0.24
2009	3/7/11	SEG	61	68	110	239	52.7	118.6	0.50	18.2	0.26
		INT	64	51	125	240	44.3	117.4	0.51	17.6	0.25
2010	3/6/12	SEG	78	58	104	240	42.6	113.7	0.45	15.8	0.19
		INT	70	66	104	240	48.5	116.4	0.53	17.3	0.26
2011	3/12/13	SEG	75	41	123	239	35.3	115.7	0.46	16.2	0.21
		INT	49	71	120	240	59.2	116.9	0.54	17.0	0.29

M = immature male, MD = age 2 developing (maturing) male, F = female.

### Part II: Common garden experiment with INT (0–1), SEG (1), and SEG (2) broodlines

Fertilized eggs were obtained from three broodlines of Yakima River spring Chinook salmon that returned to the river as adults in the spring-summer of 2007 (BY 2007). At the time of this experiment, the CESRF was rearing its third generation in culture. Thus, unmarked adults that returned to the RAMF had one generation of potential integrated hatchery culture [progeny hereafter INT (0–1)]. Marked non-SEG broodline adults that returned to RAMF in 2007 had one generation of known segregated hatchery culture [progeny hereafter SEG (1)]. Finally, based on marks and tags, the SEG broodline had exactly two generations of segregated hatchery culture at the time of the experiment [progeny hereafter SEG (2)]. A mixture of eggs was obtained from each broodline depending on availability as part of other ongoing studies [42, 46, 47] and with an emphasis on maximizing representation across multiple families as follows: INT (1)-2000 eggs from 80 families (25 eggs/family), SEG (1)-approximately 4000 eggs from 45 potential half-sib families constructed from 15 females and 5 males. The specific protocol for the SEG (1) line was to obtain 300 eggs from each female, divide them into 100 egg lots, and fertilize each lot with four drops of milt from three different males. SEG (2) broodline-3100 eggs from all 31 families that returned to RAMF in 2007 (100 eggs/family).

### Fish rearing

The egg lots from the three broodlines were transported at the “eyed” developmental stage to the Northwest Fisheries Science Center (NWFSC), Seattle, WA on November 9, 2007 and incubated in three separate egg trays at 5°C until the time of ponding on March 20, 2008. Fry were ponded (400-414/rep.) in quadruplicate 1.4 m diameter circular tanks under ambient photoperiod supplied by a closed water recirculation system with biofiltration, ozonation and ultraviolet sterilization. Water temperature ranged from 8–10°C throughout the study. Fish were reared according to standard hatchery practices using Bio Vita fish feed (Bio-Oregon, Longview, WA) under a seasonally adjusted growth regime designed to achieve moderate growth in all tanks (total = 12 tanks; 4 tanks per broodline). Growth was regulated by ration manipulation to achieve a mean smolt size of approximately 30 g in April 2009 which is similar to the standard production size achieved by the CESRF [22, 43]. Size was monitored approximately monthly by conducting batch weights of fish from each tank (20–50 fish per batch depending on date/size in three unique batches per tank). These data were used for tracking growth and adjusting rations. Fish were reared until April-May 2009 (20 months post fertilization), an age by which immature smolts and maturing age-2 minijacks are easily identifiable by the difference in testicular weight relative to body weight according to the following formula: gonad weight (g)/body weight (g) × 100 = (gonadasomatic index: GSI) [22, 28].

### Sampling

At the termination of the experiment, all fish were euthanized using a buffered solution of 0.05% MS-222 prior to handling. Fish were measured for fork length and weight. Sex and testes weights were determined according to the method of Larsen et al. [28]. If any fish matured as an age-1 microjack (from the previous September) they were easily identifiable by the presence of relatively large whitish/grey testes typical of the post-maturation resorption process we have previously observed in Chinook salmon [29]. The few age-1 microjacks that were observed survived maturity at age 1 until the time of experimental termination because none of the mortalities observed prior to the end of the study were age-1 microjacks based on postmortem dissection. Due to the significant number of fish reared and screened in this experiment, final termination of the study required processing one to two days per week over a five week period beginning April 21 through May 27, 2009. This protracted sampling period had no effect on the final determination of age-2 minijack rates since the physiological decision to mature would have been set by this time and no further recruitment to the age-2 minijack life history is physiologically possible by this late date [22, 28]. However, the protracted sampling did impact final size-at-age calculations due to the fact that the fish continued to be fed and grow over the final month of rearing. To account for expected growth and to balance the change in tank density as fish were removed for sampling, approximately equal numbers of fish were removed from each tank on each sampling date (75–100 fish). Overall growth rates over the final five weeks were calculated for each broodline by simple linear regression and found to not be significantly different (*F*_2,54_ = 0.72, *P* = 0.49). An average growth rate of 0.17 g/day was estimated over this short period of time for all broodlines and used to calculate a growth rate corrected final body weight for all fish in the study.

### Age-2 minijack rate determination

Maturation status was determined visually during the first four terminal sampling dates since testes were sufficiently developed in age-2 minijacks by late April. On the final sampling date (May 27, 2009), testes from all males were weighed to the nearest milligram to calculate GSI and confirm the accuracy of visual determination applied in the first four weeks of sampling. The GSI of all males was log_10_ transformed to detect bimodality in the distribution of the data according the method of Larsen et al. [22, 28]. All males with a GSI below and above 0.1 (log_10_ GSI = -1) were categorized as immature and maturing, respectively (Fig 2B). The final body weight, fork length and proportion of immature males, females and males initiating age-2 minijack maturation were determined for each rearing vessel.

### Age-3 jack contribution to broodstock vs. age-2 minijack rate

Previous studies on Chinook salmon have shown age at maturation to be heritable and influenced by the age of sires [40, 48]. To determine if there was any relationship between the proportion of age-3 jacks used for the CESRF broodstock and age-2 minijack rate and PMRN W_*P*50_ we estimated the proportion of eggs fertilized each year using milt from age-3 jack males. All returning adults to the Upper Yakima River were enumerated and identified according to broodline daily at the RAMF (Fig 1). The sex, age and fork length of all returning adults was determined over the course of the investigation via a combination of tags, fork length and scale analysis as described in Fast et al. [17]. Broodstock were collected proportionally throughout the adult run and the proportion of eggs artifically fertilized with milt from age-3 jacks varied across years ([17]; S1 Table). Starting in 2000, a goal of 10% progeny sired by age-3 jacks was used for the FNDR/INT broodline whereas the SEG broodline often fertilized a higher proportion of eggs with milt from age-3 jacks due to lower availability of hatchery-origin males in the broodstock.

### Statistical analysis

#### Broodline differences in size

Mean body weights were compared for both Parts I and II to make sure the two populations experienced similar growth. For Part I, the body weight of individual males at the time of maturation assessment (March) was determined by broodline for each brood year separately using *t*-tests. Overall differences across brood years were assessed by one-way ANOVA with brood years as replicates. In Part II, batch weights (these estimates include females) that were conducted approximately monthly were compared among broodlines by two-way ANOVA (tanks as replicates) with the Bonferonni correction for multiple comparisons to adjust significance levels; and weight of males during final maturation assessment by one-way ANOVA.

#### Broodline differences in age-2 minijack rate and PMRN

Two approaches were used to assess incidence of early male maturation. The first and simplist was the comparison of early male maturation rates [percent maturation among males; age-1 microjack (Part II only) and age-2 minijack] between broodlines. Maturation rates were assessed by beta regression with brood years as replicates for Part I and tanks as replicates for Part II. The second approach was to use logistic regression analysis (PMRN) to compare probability of maturation among broodlines. Logistic regression analyses was more powerful as it captured the variation in body weight and probability of maturation of individual males within broodline and brood year. In Part I, data from the FNDR/INT broodlines represented a continuum of replicate years over time and were combined for comparison to that of the SEG broodline. In Part II, the regression analysis compared the probability of maturation for the INT(0–1), SEG (1) and SEG (2) broodlines. The PMRN analysis was not conducted for the age-1 microjack phenotype observed in Part II due to insufficient numbers of fish expressing that phenotype and the fact that it should be done prior to final maturation rather than after the fish have fully matured.

The incidence of age-2 minijack maturation is a binary response variable, so the relationship between size and probability of age-2 minijack maturation among males (PMRN) can be determined using the logistic regression model

$$\mathrm{logit}[p(m)]=\beta_{0}+\beta_{1}w$$

where *m* is the probability of age-2 minijack maturation, β_0_ is the coefficient estimate for the constant and β_1_*w* is the coefficient estimate for body weight. Next, the predicted body weight at 50% age-2 minijack maturation (PMRN W _*P*50_) can be determined from these parameter estimates from each logistic regression model using the following equation:

$$\mathrm{PMRN}\;W_{P50}={-\beta }_{0}∕\beta_{1}w$$

This represents the mean body weight where 50% of the males are maturing as age-2 minijacks and is the midpoint of the PMRN. For our analyses (both Part I and Part II), broodline was included in the logistic regression models that were used to predict PMRN W_*P*50_ (separate models were run for each brood year to derive this estimate in Part I, see S1 Text for coding in STATA).

There are two ways of comparing PMRNs of different broodlines for a given age class. First, by including the factor variable of broodline (*g*) in the logistic regression model;

$$\mathrm{logit}[p(m)]=\beta_{0}+\beta_{1}w+\beta_{2}g$$

and in the case of the Part I comparison, also adding the factor variable brood year (*y*) and its interaction with *g* to take into account the effect of brood year.

$$\mathrm{logit}[p(m)]=\beta_{0}+\beta_{1}w+\beta_{2}g+\beta_{3}y+\beta_{4}gy$$

Second, by comparing the maturation index estimates, PMRN W _*P*50_, and its estimated SEM from each broodline by ANOVA (Part I using two-way ANOVA including the factor brood year and its interaction with broodline, Part II using one-way ANOVA with Tukey’s multiple comparisons test).

#### Differences in weight/growth and assessing age-2 minijack rate and PMRN

**Fig 3:**
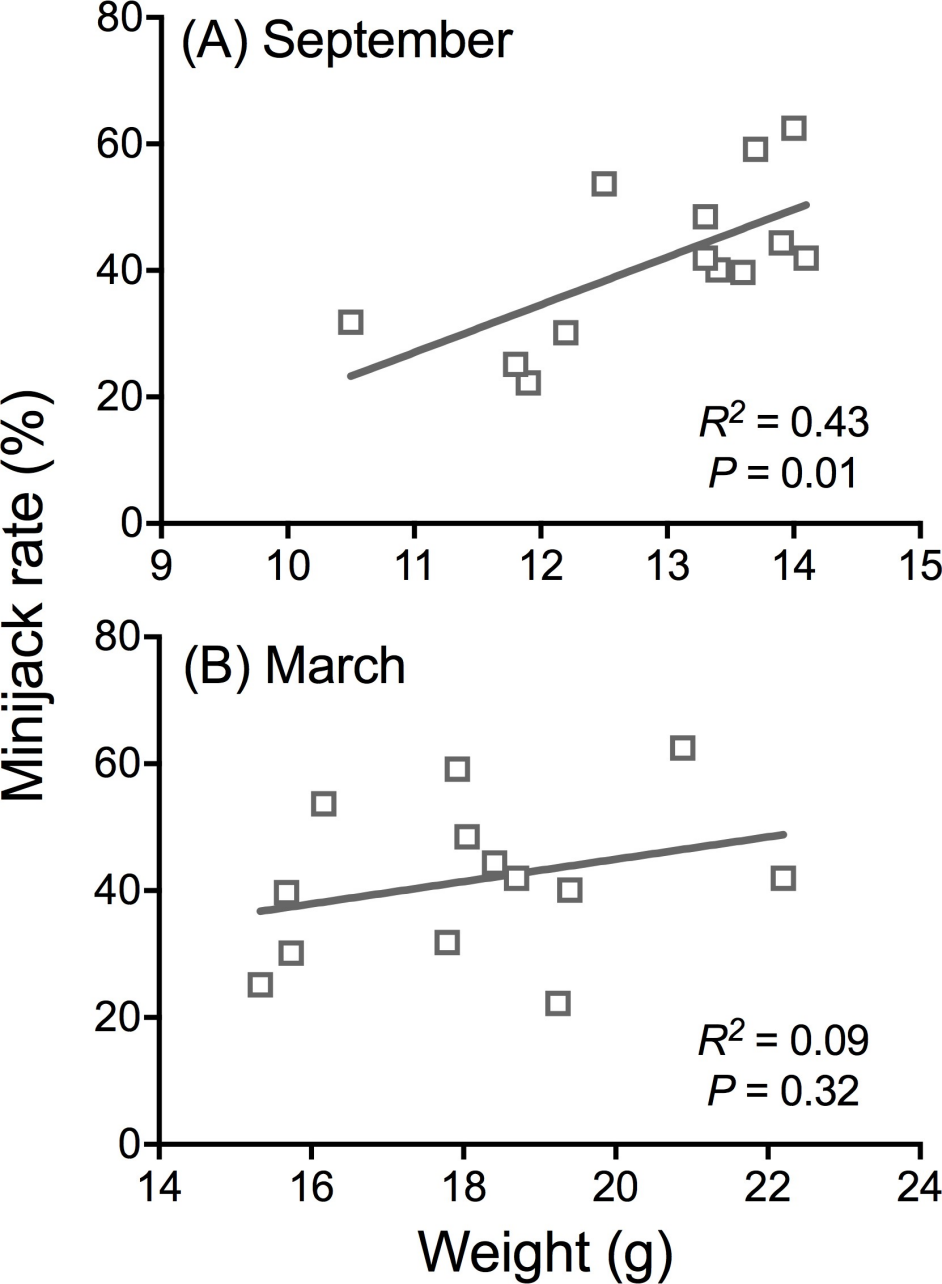
Seasonal relationships between mean fish weight and age-2 minijack rate (percent among males). **(A)** September vs. **(B)** March. FNDR and INT broodlines from BYs 1998–2011 were used for the linear regression analyses. The outliers (BYs 1998 & 2004) were omitted due to large difference in fall size/growth rate of these rearing years from all other years.

The physiological “decision” to mature in spring Chinook salmon is made in the summer-fall period approximately 9–12 months prior to spermiation for each age class [43, 49]. Comparing size thresholds (PMRN W_*P*50_) estimates can be problematic if growth is not equal among groups being compared from the time of the maturation decision until the date the size measurements of the individual fish are taken. For Part I, linear regression analysis showed that weight in September (Fig 3A) was a better predictor of age-2 minijack rate than at release in March (Fig 3B). Also differences in winter growth rate existed between broodlines and brood years (see S2 Table). To address this, we calculated adjusted PMRN W_*P*50_ estimates for each release group using population batch weights (WT) taken by hatchery staff at CESRF to track ration and growth rate throughout rearing.

$$\mathrm{Adjusted}\;\mathrm{PMRN}\;W_{P50}=\mathrm{PMRN}\;W_{P50}-({\mathrm{WT}}_{Release}-{\mathrm{WT}}_{September})$$

This adjustment improved the relationship between the predicted threshold based on weight and age-2 minijack rate (S1 Fig). BYs 1998 and 2004 were outliers in rearing size. They were smaller during their first June (1.0 g and 1.5 g, respectively) than all other brood years studied (range: 2.1–3.6 g; S2 Table). They experienced higher growth rates going into their first fall and subsequently had higher age-2 minijack rates than would be predicted by their weight the following fall. Thus, they were removed from regression analysis of fall weight and age-2 minijack rate. Growth rates were the same for each treatment in Part II which made adjusting PMRN W_*P*50_ estimates unneccessary.

#### Differences in age-3 jack returns and contribution to broodstock

The relationship between the proportion of eggs that were fertilized with milt from age-3 jack males and subsequent age-3 jack returns and age-2 minijack rates among cohorts for the FNDR/INT and SEG broodlines was analyzed using beta regression. We then examined potential differences between the relationship between age-3 jack contribution and age-2 minijack rates and adjusted PMRN W_*P*50_ for the FNDR/INT and SEG broodlines using simple linear regression.

All data analyses and graphics were completed using a combination of STATA/IC version 15.1 (StataCorp LP, College Station, TX) and GraphPad Prism version 7 (GraphPad Software Inc., La Jolla, CA) software. Statistical significance was set at a level of α = 0.05. The variables ‘broodline’ and ‘brood year’ were treated as categorical variables in all analyses; the variables ‘body weight’, ‘maturation rate’ (age-1 micojack and age-2 minijack), and ‘PMRN W_*P*50_’ were treated as continuous variables in all analyses.

## Results

### Part I: Comparing body weight, age-2 minijack rates and PMRN of progeny from FNDR, INT and SEG broodlines at Clark Flat acclimation site

#### Body Weight and age-2 minijack rate

**Fig 4:**
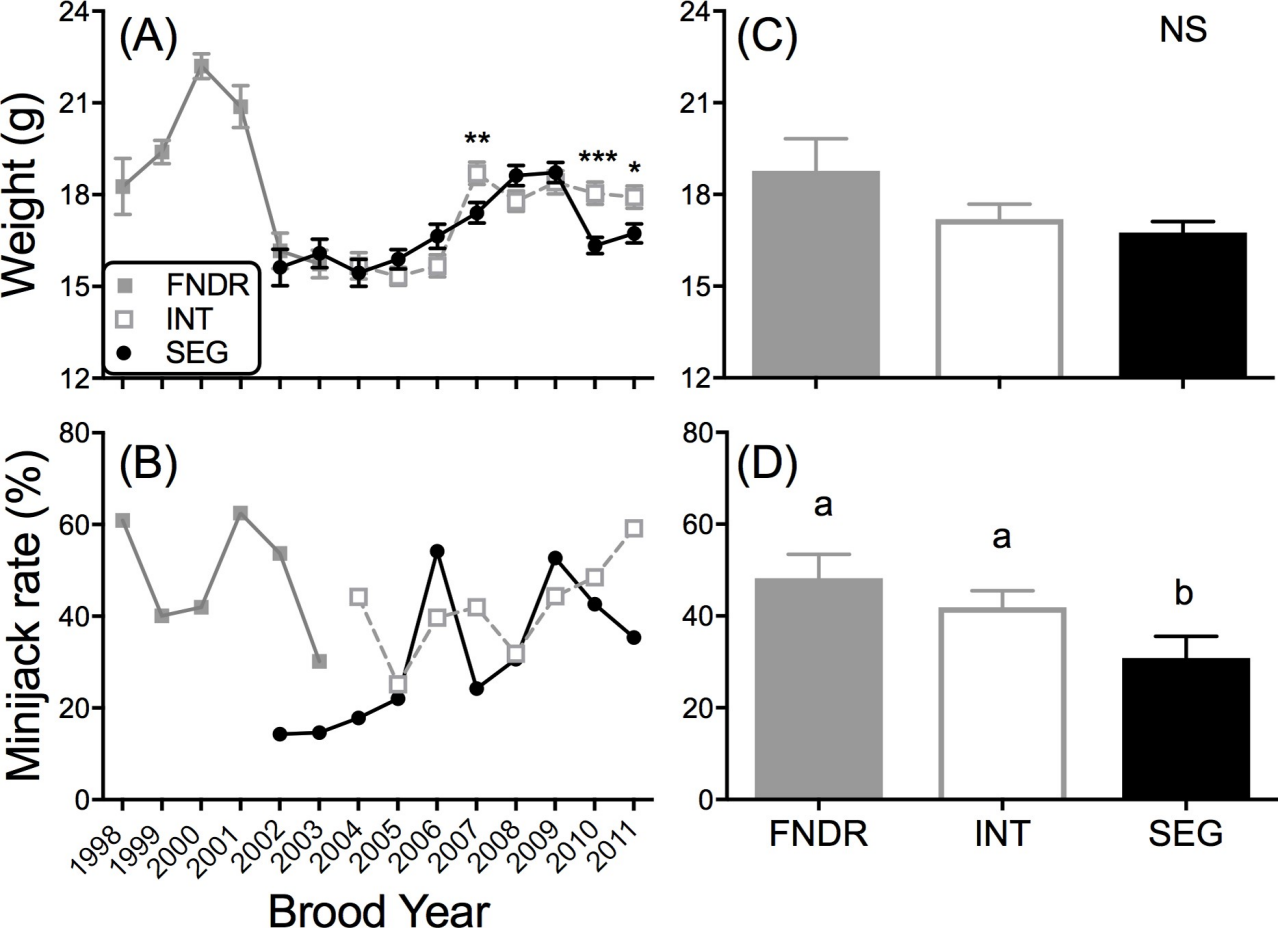
Year-to-year variation in body weight at release and age-2 minijack rate at Clark Flat acclimation site. (**A)** Mean body weight (g) of males at release and **(B)** percent age-2 minijacks among males for BYs 1998–2011; and the across brood year averages for each broodline for **(C)** body weight and **(D)** age-2 minijack rate. The founding hatchery line (FNDR) was produced from wild-origin broodstock (BYs 1998–2003). The integrated (INT) broodline began in 2004 and uses only unmarked (natural-origin; could have hatchery-reared grandparents) returning adults for broodstock. The segregated (SEG) broodline began in 2002 using returning hatchery-origin adults for broodstock. Error bars = SEM. Different letters above bars represent a significant difference between broodlines (α = 0.05). *P < 0.05, **P < 0.01, ***P < 0.001.

Mean body weight at release varied significantly ranging from 15.3 to 22.2 g across all broodlines and brood years (Fig 4A). But, body weight was more comparable within brood year between genetic lines (SEG vs. FNDR, SEG vs. INT) with just three of ten brood years (BYs 2007, 2010, and 2011) having statistical differences in mean body weight between genetic lines (*P* < 0.05, Fig 4A). There was no significant overall difference in mean body weight across brood years between the progeny of the FNDR broodlines (*F*_2, 21_ = 2.852, *P* > 0.05, Fig 4C). With the exception of BYs 2006 and 2009, age-2 minijack rates in the progeny of the FNDR and INT broodlines were higher than that of the SEG broodline (Fig 4B). Significant differences in mean age-2 minijack rates (± SEM) across brood years were found between progeny of the different broodlines [likelihood ratio (LR) χ^2^ = 7.69, df = 2, *P* = 0.02, Fig 4D]. The age-2 minijack rates of the progeny of the FNDR and INT broodlines were not significantly different (*z* = -0.91, *P* > 0.05), but the progeny of the SEG broodline had signficantly lower age-2 minijack rates compared to progeny of the FNDR (*z* = -2.84, *P* = 0.004) and INT (*z* = -2.06, *P* = 0.04) broodlines (Fig 4D).

**Fig 5:**
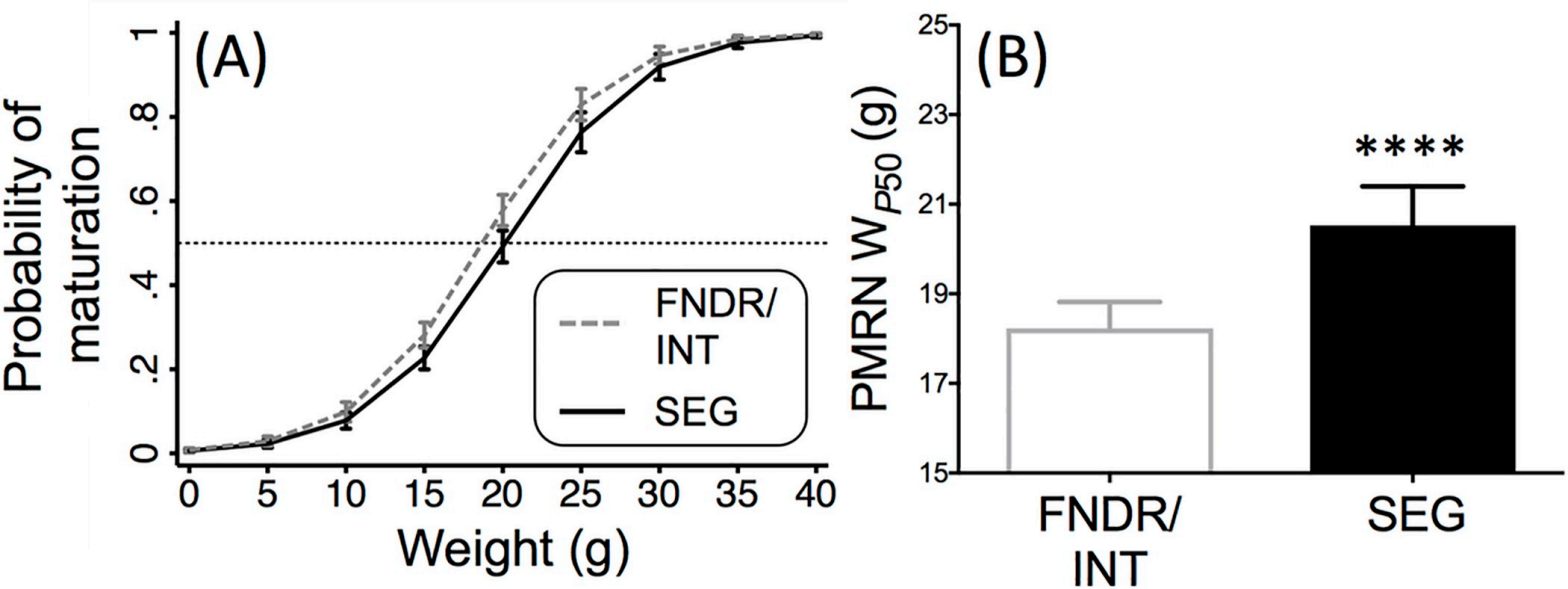
**Reaction norm (A) and PMRN W**_**P50**_ **(B) estimates, Part I. (A)** Marginally predicted probabilities of age-2 minijack maturation with 95% CI based on the following model: logit[*p*(*m*)] = β_0_ + β_1_*w* + β_2_*g* + β_3_*y* + β_4_*gy* (*w* = weight, *g* = broodline, *y* = brood year) for spring Chinook salmon sampled from Clark Flat acclimation site, BYs 2002–2011 (see S3 Table). Dashed horizontal line indicates probability of 50% maturity. **(B)** Mean weight at 50% maturity (PMRN W_*P*50_) estimates, years as replicates{estimated from the model: logit[*p*(*m*)] = β_0_ + β_1_*w* + β_2_*g*; run separately for each brood year (see S4 Table)}. Only males were included in these analyses. **** *P* < 0.0001.

Logistic regression analyses compared the FNDR/INT broodline to that of the SEG broodline and found body weight at release, brood year, and broodline to all be significant predictors of incidence of age-2 minijack maturation among males (S3 and S4 Tables). First, having a greater weight at release increases the probability of age-2 minijack maturation among males (Odds ratio = 1.3, *z* = 16.61, *P* < 0.001, S3 Table). Analyses for each brood year separately found broodline also to be a significant predictor of maturation in five of the ten study years (S4 Table), with the FNDR/INT broodline having a greater probability of maturation for a given weight than the SEG broodline in those five years. In addition, the full logistic regression model that included brood year as a factor found the odds of age-2 minijack maturation to be 1.37 times higher for the FNDR/INT broodline compared to SEG broodline (Odds ratio = 1.37, *z* = 3.14, *P* = 0.002, S3 Table and Fig 5A). Brood year, and its interaction with broodline, were also significant (S3 Table) showing again that there was some yearly variation in the relationship between maturation and broodline. The mean PMRN W_*P*50_ ± SEM was significantly different between the SEG (20.53 ± 0.87 g) and FNDR/INT broodlines (18.23 ± 0.59 g) (*F*_1, 2074_ = 18.57, *P* < 0.0001, Fig 5B).

#### Cohort specific trends in age-2 minijack rate and PMRN W_*P50*_

**Fig 6:**
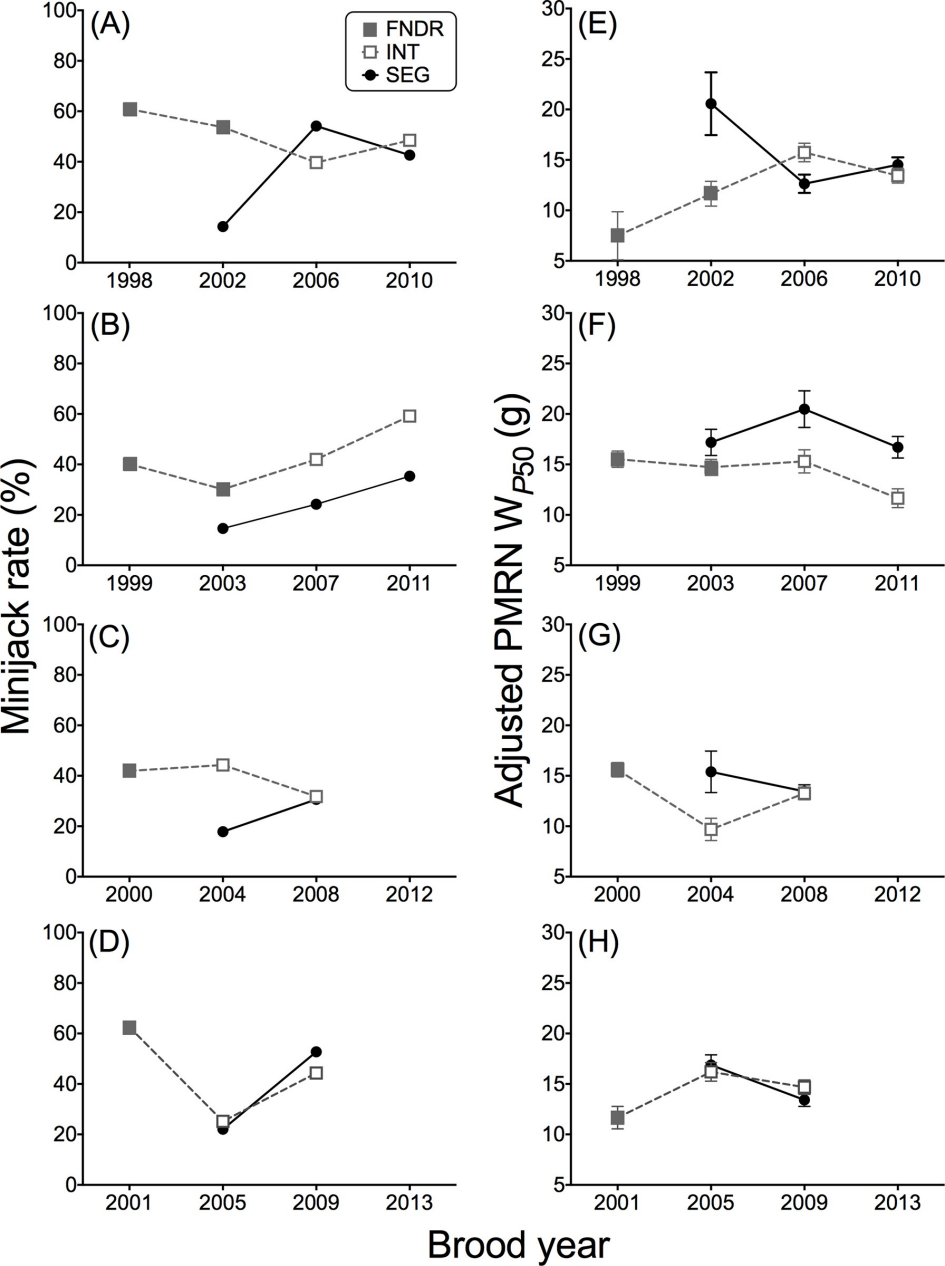
Across generation examination of age-2 minijack rate and PMRN W_P50_ estimates. **(A-D)** Age-2 minijack rates and adjusted PMRN W_*P5*0_ estimates with SEM **(E-H)** by broodline across generations for the founding (FNDR) brood years (BYs 1998–2001). A majority of the spawning Chinook salmon returning to the Yakima River are age-4 adults, thus generation time for each cohort is approximately 4 years. See Fig 4 for FNDR, INT, SEG definitions.

The majority of adult fish return to the Yakima River at age 4, so cohort specific trends in age-2 minijack rate could be examined every four years when progeny return as adults. Age-2 minijack rates were determined across four FNDR cohorts (BYs 1998–2001, Fig 6A–6D). With the exception of the 1999 FNDR broodline there was no consistent pattern among cohorts or broodlines. The age-2 minijack rate of the 1999 FNDR broodline was 40% and rates ranged from 30% (2003 FNDR), 42% (2007 INT) and 59% (2011 INT) (Fig 6B). By comparison the age-2 minijack rates of the corresponding 2003, 2007 and 2011 SEG broodlines were consistently lower than that of the INT broodline ranging from 15%, 24% and 35%, respectively (Fig 6B).

The cohort specific adjusted PMRN W_*P*50_ was tracked throughout the investigation (Fig 6E–6H) and, as with the age-2 minjack rates, the clearest separation between the FNDR/INT and SEG broodlines was observed in the BY 1999 cohort (Fig 6F) where the adjusted PMRN W_*P*50_ was relatively consistent, ranging from 11.0–15.7 g. The adjusted cohort specific PMRN W_*P*50_ of the SEG broodline (BYs 2003, 2007, and 2011) was consistently higher than that of the FNDR/INT broodline [Paired *t*-test (paired by brood year): *t* = 4.76, df = 2, *P* = 0.04]. By contrast, the adjusted PMRN W_*P*50_ of the BY 1998 (Fig 6E), BY 2000 (Fig 6G), and BY 2001 (Fig 6H) cohorts were variable with no clear rank order between the FNDR/INT and SEG broodlines.

#### Age-3 jack contribution to cohort age-3 jack and age-2 minijack rates

**Fig 7:**
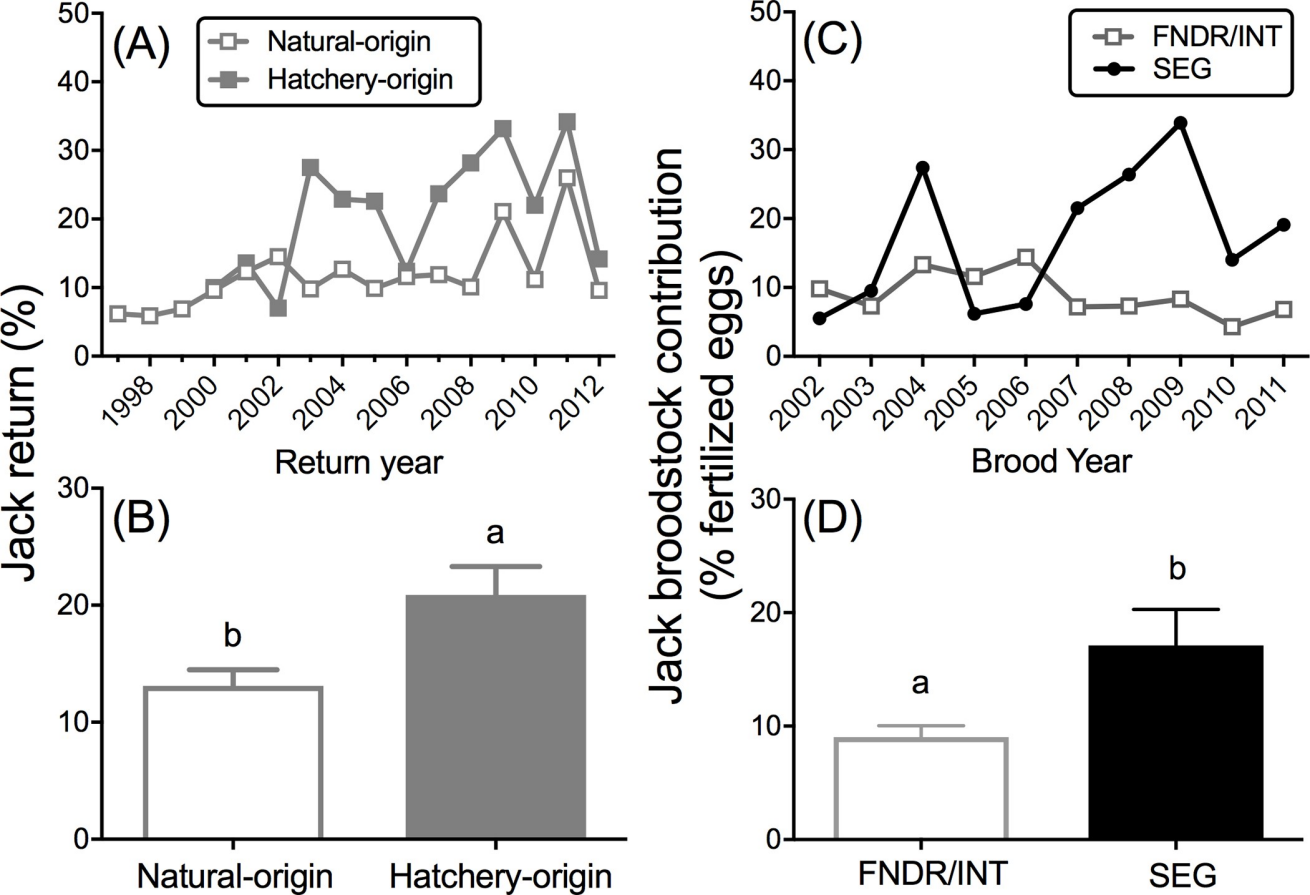
Age-3 jack return rates and contribution to hatchery broodstock. (**A**) The percent of natural- and hatchery-origin age-3 jacks (among males and females) that returned to RAMF during 1997–2012 (among their release cohort, age 4 and age 5 fish returned in the following years) and **(B)** averaged over return years 2000–2012. **(C)** The estimated percentage of eggs fertilized by age-3 jack males from the natural-origin (FNDR, INT) and hatchery-origin (SEG) broodstock during BYs 2002–2011 at CESRF and **(D)** averaged over BYs 2002–2011. Different letters within figure represent statistical differences, α = 0.05.

Marked and unmarked age-3 jack males returned to RAMF at variable rates (among males and females combined) according to brood year (Fig 7A) with unmarked fish (reared in the natural environment as juveniles) having lower mean age-3 jack rates than the marked fish [beta regression (BYs 2000–2012): χ^2^ = 6.77, df = 1; *P* = 0.009; Fig 7B]. The proportion of eggs that were fertilized by age-3 jacks at CESRF varied across brood years (Fig 7C), and was signifcantly different between FNDR/INT and SEG broodlines [beta regression (BYs 2002–2011): χ^2^ = 4.40, df = 1, *P* = 0.034, Fig 7D; averaging 9.0% ± 1.0%, and 17.1% ± 3.2% in the FNDR/INT, and SEG broodlines, respectively]. There was a positive (but, not significant) trend between age-3 jack contributions in matings and age-2 minijack rates (*R*^2^ = 0.33, *P* = 0.11) in the SEG broodline (BYs 2002–2011) when the outlier year BY 2006 was removed (including BY 2006: *R*^2^ = 0.073, *P* = 0.45). There was no relationship observed in the FNDR/INT broodline (*P* = 0.53), but there was also less variation in the rate of age-3 jack contribution compared to the SEG broodline (S2 Fig).

### Part II: Common garden experiment

Mortality was highest in the first two months after ponding, averaging 3.8% among all tanks and decreased to less than 1% throughout the remainder of the experiment. Due to an error in counting of fish into one of the four SEG broodline tanks during ponding, these fish experienced higher density and lower average growth throughout the experiment. Thus, this replicate was omitted from all analyses.

#### Body weight and growth

**Fig 8:**
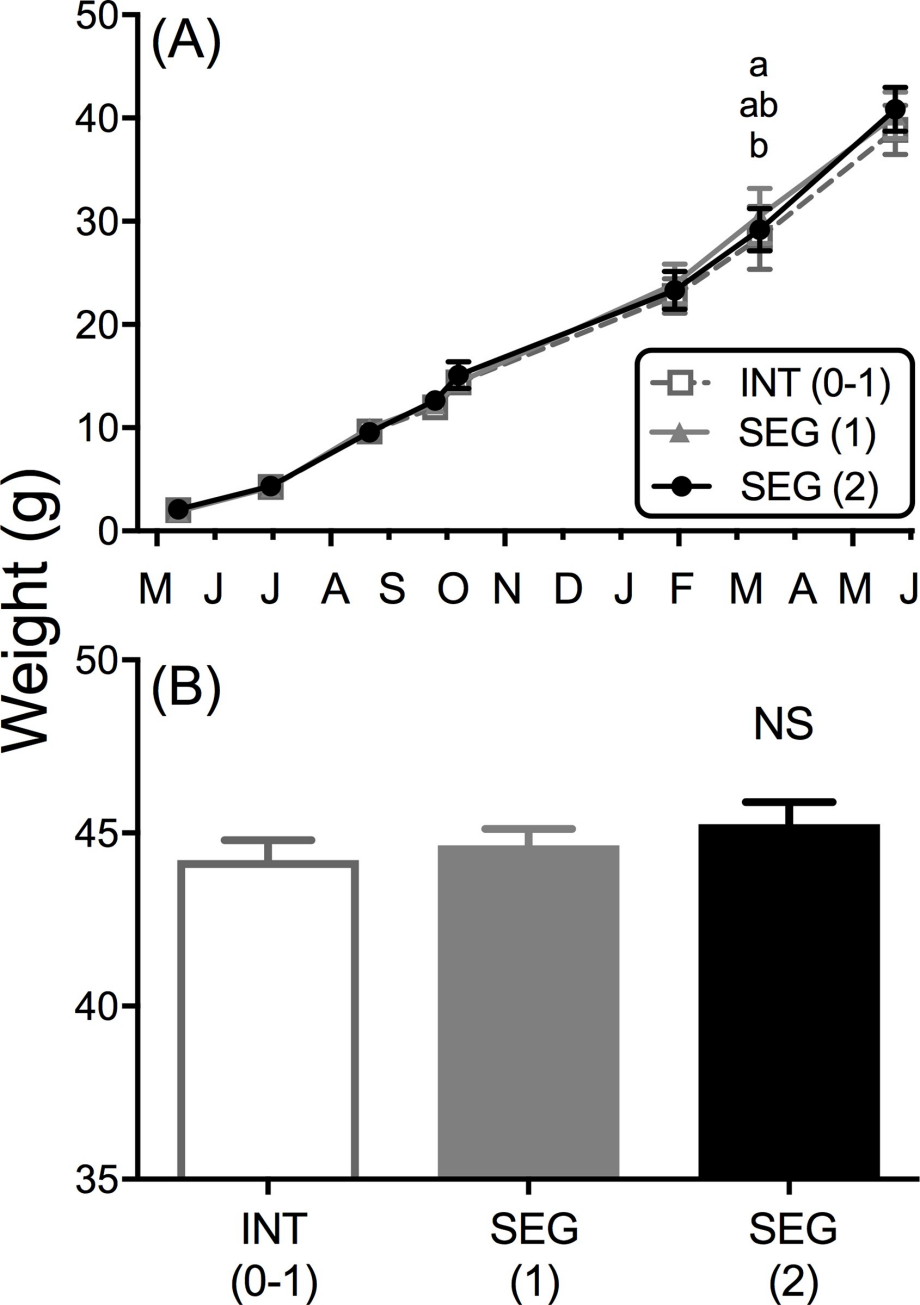
Growth profile comparison by broodline for common garden experiment (Part II). **(A)** Mean body weight (g) across dates during the experiment based on batch weights (*N* ranged from 12 to 16 per treatment) for all dates except the final sample where mean weight of each tank was used as replicates (*N* ranged from 3 to 4). Females are included in estimates as sex of fish was not known during batch weights. Different letters represent significant difference between broodline treatments, α = 0.05. **(B)** Mean adjusted body weights of individual males at completion of experiment (*N* ranged from 571 to 735 per treatment). Error bars = SEM.

All three broodlines experienced nearly identical growth over the course of the experiment with only one sample date, March 18, 2009, having any statistical difference between broodlines [SEG (1) > INT (0–1); mean difference = 2.12 g, *t* = 4.085, *P* < 0.01, Fig 8A]. Examination of weight of each broodline at the termination of the experiment (the weeks of April 27 –May 27, 2009) found no significant differences in the “growth rate corrected” final mean weights (*F*_2, 2035_ = 0.818, *P* > 0.05, Fig 8B). Therefore equal growth can be assumed among treatments.

#### Age-1 microjack maturation

**Fig 9:**
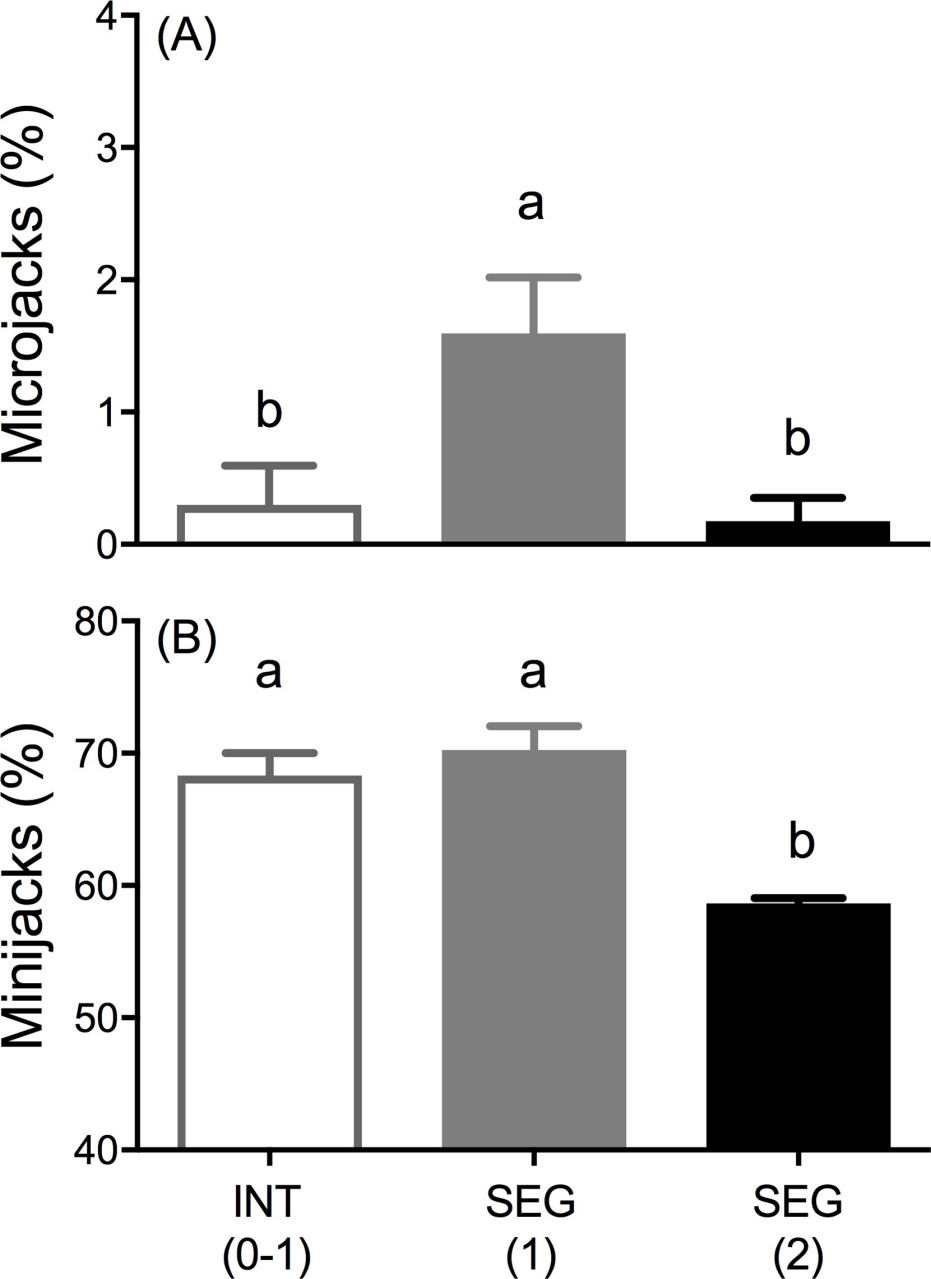
Early male maturation rates of broodline treatments in the common garden experiment (Part II). **(A)** Percent age-1 microjacks among males. **(B)** Percent age-2 minijacks among males. Tanks are replicates (*N* ranged from 3 to 4 per treatment). Different letters within panel represent significant differences between broodline treatment using beta regression analyses, α = 0.05. Error bars = SEM.

A small percentage of the males in each treatment matured as age-1 microjacks and rates varied significantly among the broodlines (LR χ^2^ = 14.39, df = 2, *P* = 0.0008) with the SEG (2) and INT (0–1) broodlines having the lowest rates of 0.18 ± 0.18% and 0.3 ± 0.3% respectively; and SEG (1) broodline having the highest rate of 1.6 ± 0.42% (Fig 9A). By contrast, age-2 minijack rates at the end of the experiment were very high, exceeding 50% of all males in each broodline (Fig 9B). Overall, broodline was a significant predictor of age-2 minijack rate (LR χ^2^ = 15.82, df = 2, *P* = 0.0004) with the SEG (2) broodline having a statistically lower age-2 minijack rate (58.63 ± 0.4%) than either the INT (0–1) (68.3 ± 1.7%) or SEG (1) (70.3 ± 1.8%) broodlines (Fig 9B).

#### Odds of maturation and PMRN W_*P50*_

**Fig 10:**
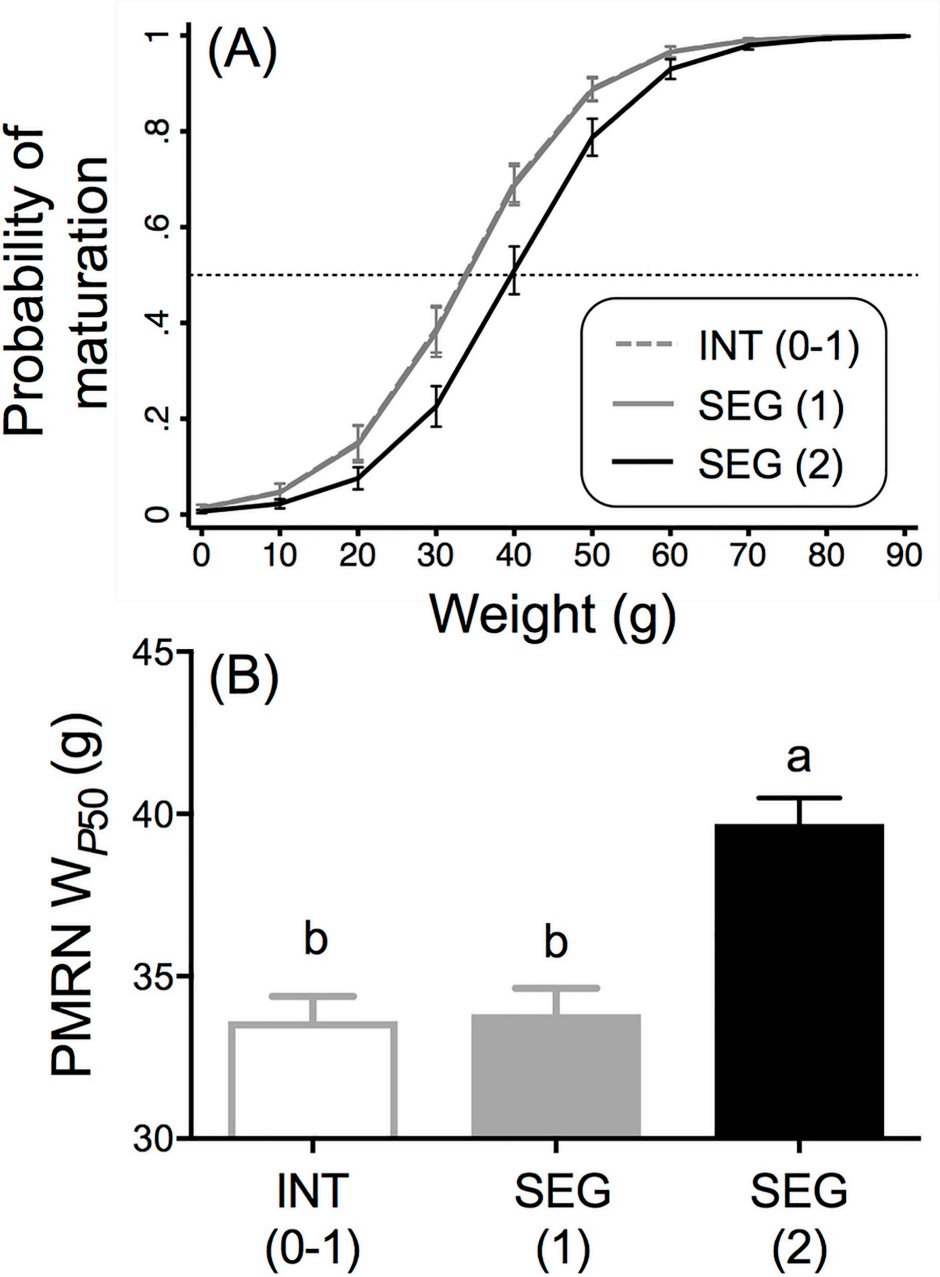
**Reaction norm (A) and PMRN W**_**P50**_ **(B) estimates from the common garden experiment (Part II). (A)** Marginally predicted probabilities of age-2 minijack maturation with 95% CI based on the following model: logit[*p*(*m*)] = β_0_ + β_1_*w* + β_2_*g* (see S5 Table). Dashed horizontal line indicates probability of 50% maturity. **(B)** Body Weight at 50% maturity (PMRN W_*P*50_) and SEM derived from the logit model in panel A. Different letters represent significant difference between broodline treatments (α = 0.05).

The logistic regression analyses for size-at-age (mean weight in late May) and incidence of age-2 minijack maturation again found both body weight and broodline to be significant predictors of the odds of age-2 minijack maturation (body weight: odds ratio = 1.14, *z* = 19.63, *P* < 0.001; broodline: *χ*^2^ = 37.62, *P* < 0.001, S5 Table). The odds of maturing increased 14% for an increase in weight of one gram. Among the broodlines, the SEG (2) broodline was signficantly different than both the INT (0–1) and SEG (1) broodlines (Fig 10A) where the odds of maturation for SEG (1) was 2.11 times greater (*z* = 5.29, *P* < 0.001) and INT (0–1) was 2.16 times greater (*z* = 5.47, *P* < 0.001) than odds of maturation for the SEG (2) broodline males. The odds of maturing among the INT (0–1) and SEG (1) broodlines were not significantly different (S5 Table). The mean PMRN W_*P*50_ for the SEG (2) broodline was significantly greater compared to the INT (0–1) (mean difference = 6.1 g, *q* = 7.4, *P* < 0.0001, Fig 10B) and the SEG (1) (mean difference = 5.9 g, *q* = 7.2, *P* < 0.0001, Fig 10B) broodlines; there was no significant difference in PMRN W_*P*50_, between the INT (0–1) and SEG (1) broodlines (Fig 10B).

## Discussion

A series of studies conducted over the past two decades have examined the causes [27, 43, 50, 51], magnitude [22, 28, 29] and implications [6, 22, 28, 43] of high rates of precocious male maturation in hatchery spring Chinook salmon. Over a seven year period, an average of 41% of all FNDR/INT broodline male fish released from the CESRF were estimated to be initiating maturation as age-2 minijacks and this rate was approximately 10-fold higher than that estimated for naturally rearing fish in the Yakima River [22]. A fundamental conclusion drawn from these earlier studies was that hatchery culture conditions can have significant effects on size and age at maturation. In the current investigation we endeavored to determine how integrated and segregated hatchery management strategies affect precocious male maturation in spring Chinook salmon. We provided two lines of evidence to support our initial hypothesis that the age-2 minijack rates and the PMRN of spring Chinook salmon may change as a result of segregated hatchery culture. Furthermore, we have attempted to determine what proportion of the variance in age-2 minijack rate and the PMRN is attributable to genetic factors (broodline, brood year and age-3 jack contribution), rearing environment (reflected in size and growth rate), as well as other potential unknown factors. Navigating this analysis with so many potential sources of variation presented some clear challenges. But, the fact that significant differences were detected, despite such variation, provides strong evidence to support our conclusions.

In Part I we found that age-2 minijack rates were significantly lower and the PMRN W_*P*50_ was significantly higher in the SEG than the FNDR/INT broodline of Yakima River spring Chinook salmon. We monitored the body weight and age-2 minijack rates (via plasma 11-KT) in progeny from the FNDR, INT and SEG broodlines over the course of 14 brood years. In BYs 2002 through 2004 we observed higher age-2 minijack rates in the FNDR/INT compared to the SEG broodline. However, from BYs 2005 to 2009 rates were variable with no clear pattern in rank order. Finally, over BYs 2010 and 2011 the INT broodline once again had higher age-2 minijack rates than the SEG broodline. Despite this interannual variability there was a significant difference in the overall mean age-2 minijack rate between the FNDR/INT and SEG broodlines. The logistic regression analysis of PMRN has some advantages over simply measuring mean age-2 minijack rates in tanks, raceways, or a population. The PMRN incorporates both the binary component of being a smolt or a age-2 minijack along with individual measure of body weight, thus increasing the diagnostic power to detect differences between the broodlines. In that analysis there was a significant (~ 10%) difference on average in the PMRN W_*P*50_, from 18.2 ± 0.6 g to 20.5 ± 0.9 g in the INT and SEG broodlines, respectively, suggesting that SEG culture may be shifting the maturation threshold to a larger size.

When we tracked the differences in age-2 minijack rates and PMRN W_*P*50_ between the FNDR/INT broodlines and SEG broodlines (BYs 1998–2001) according to age-4 cohort, consistent differences were only observed in BY 1999. In BY 1999, the FNDR/INT broodline had significantly higher age-2 minijack rates than the SEG broodline across subsequent brood years for that cohort. However, this same rank order was not observed for the BY 1998, 2000, or 2001 cohorts. These results suggest that there may be cohort specific differences in early male maturation in some brood years (BY 1999), but not others. The source of that variation may be family specific or due to some other factors we did not measure.

The clear differences between the SEG and INT broodlines observed during the first few years of monitoring described in Part I inspired the initiation of Part II. In Part II we used a common garden rearing experiment to compare minijack rates and the PMRN W_*P50*_ of SEG broodline offspring that experienced one or two generation(s) in culture with that of INT broodline offspring that experienced zero or one generation in culture.

While age-2 minijack rates were relatively high in all three broodlines (rates of wild fish have been estimated to be approximately 10-fold lower than that of hatchery fish [22]), they were significantly lower in the SEG (2) than the SEG (1) and INT (0–1) broodlines. It is noteworthy that during our monitoring effort in Part I at the CESRF that 42% and 24% of all male progeny sampled from BY 2007 were age-2 minijacks in the INT (0–1) and SEG (2) broodlines, respectively. But, when these same BY 2007 INT (0–1) and SEG (2) broodlines were reared at the NWFSC for Part II of this investigation age-2 minijack rates averaged 68.3% ± 1.7% and 58.6% ± 0.4%, respectively [the age-2 minijack rate of the SEG (1) broodline in Part II was 70.3 ± 1.8%, but not monitored at the CESRF in Part I]. While efforts were made to match the growth profiles of our common garden fish to that of the CESRF, the water temperatures at the CESRF and the Clark Flat acclimation site vary seasonally from a low of 1°C in January to a high of 15°C in August [28]. By contrast the water temperature in the water recirculation system at the NWFSC averages 8–10°C throughout the year. The potential for growth and associated propensity for precocious male maturation was higher in Part II and even though the rates were high in all broodlines, significant differences were observed. Similar to the aformentioned age-2 minijack monitoring effort in Part I, the PMRN W_*P*50_ was significantly higher in the SEG (2) (39.7 ± 0.8 g) than the SEG (1) (33.8 ± 0.8 g) and INT (0–1) (33.6 ± 0.8 g) broodlines; a 6.1 g increase from INT (0–1) to SEG (2). Taken together, these two lines of inquiry demonstrated that the PMRN for age-2 minijack maturation in Yakima River spring Chinook salmon has shifted to a significantly larger body size threshold after as little as two generations of segregated hatchery culture and provides some supportive evidence of contemporary evolution in this important life history attribute [52, 53, 54, 55].

**Fig 11:**
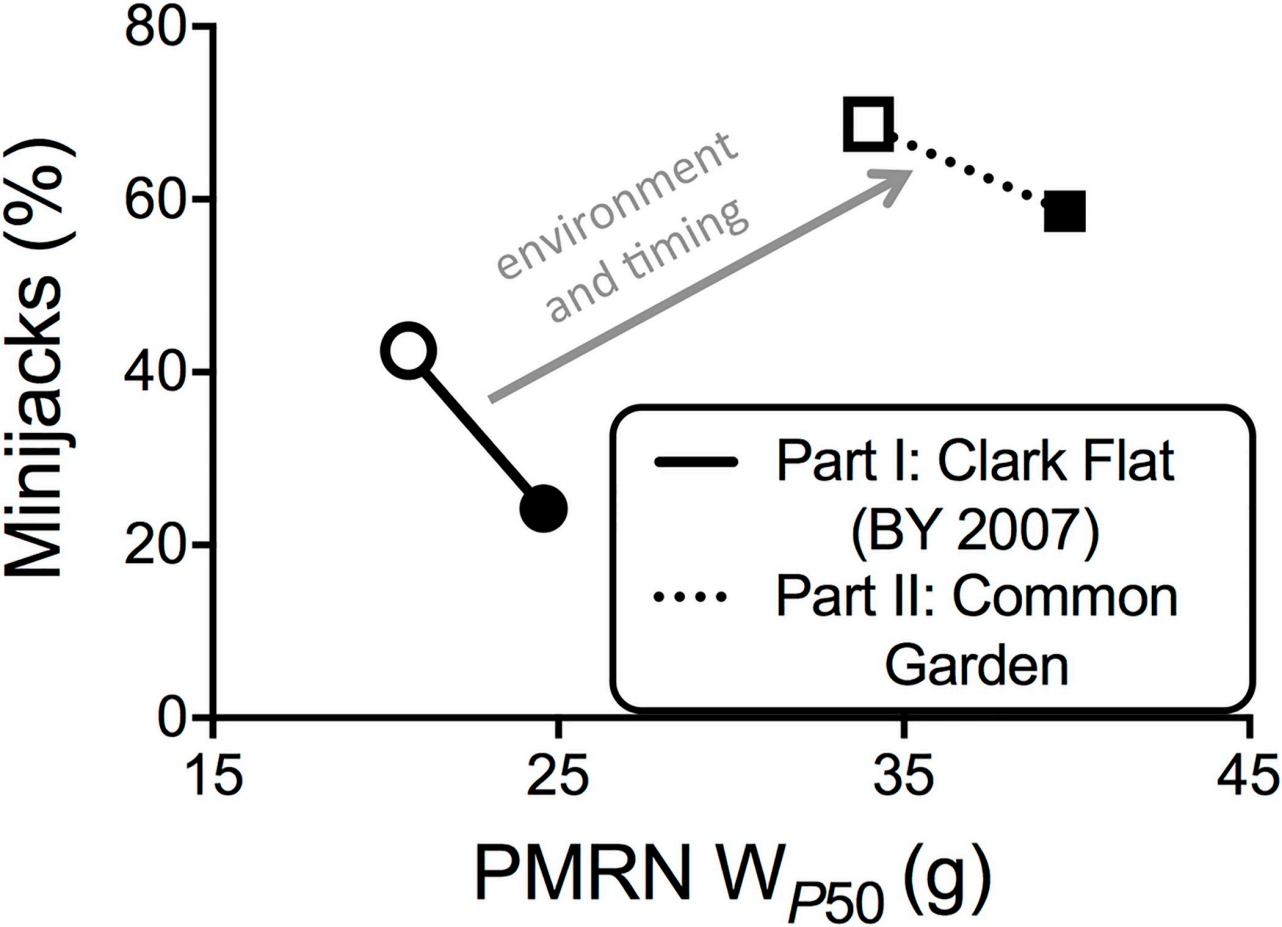
Comparison of age-2 minijack rate and PMRN W_P50_ estimates from Part I and Part II. Relative difference in the relationship between the mean age-2 minijack rate and mean PMRN W_*P*50_ of the integrated/founder (INT/FNDR) and segregated (SEG) broodline from Part I and Part II of this investigation; both groups presented were progeny of BY 2007 broodstock. Open symbols = INT; closed symbols = SEG.

When comparing differences in the PMRN W _*P*50_ between Part I and Part II, we found clearly different values. For example the PMRN W _*P*50_ of the SEG (2) broodline was 24.6 ± 1.8 g in Part I and 39.7 ± 0.8 g in Part II (Fig 11). This is a result of variation in the age at which the fish were measured, water temperature, and potential differences in feed rate. In Part I the fish were measured in March, approximately 3–5 months after the “physiological decision” to mature, and approximately six months prior to spawning in September [49]. In Part II they were measured in May, approximately 5–7 months after the decision and four months prior to spawing in September. As discussed in Spangenberg et al. [56], the threshold analysis doesn’t provide an absolute size for the maturation decision, but does allow one to compare relative sizes among individuals that were reared under the same growth regime at a point in development when immature and maturing males can be differentiated via simple visual inspection, GSI, or plasma11-KT levels [28], but prior to major changes in body size associated with the final maturation process [49].

Previous studies have demonstrated that fertilization of eggs with milt from age-3 jack males increases the probability of their male progeny maturing as age-3 jacks [40, 48]. Hankin et al. [40] found over two years of hatchery releases that crosses between age-4 females and age-2 jack male fall Chinook salmon (comparable to age-3 jacks in spring Chinook salmon) resulted in return rates of 60% and 76% jacks in subesequent years while crosses between age-4 females and age-4 males resulted in return rates of only 7.6% and 25%. This genetic component to age of maturation was also examined in the current investigation by examining the effect of the proportion of eggs potentially fertilized by milt from age-3 jacks on age-2 minijack rates in their offspring. In Part I of this study, a positive (but not significant) trend in this relationship was observed for the SEG broodline, but not the INT broodline. However, on average, a greater percentage of SEG broodline eggs were fertilized with milt from age-3 jacks, but the age-2 minijack rates of SEG broodline were, in fact, significantly lower than that of the INT broodline. One pairwise comparison (within brood year) of note was BY 2009. Age-3 jack contribution was estimated at 33.9% in the SEG broodline but just 8.3% in the INT broodline in that brood year. Subsequently, this was one of the two years in which age-2 minijack rates in the SEG broodline exceeded those in the FNDR/INT broodline in 11 years of this study. Taken together, we found no consistent evidence to definitively suggest variation in age-3 jack contribution affected age-2 minijack rates of their progeny, but it may have added to some of the variability we observed.

Various phenotypic traits have been monitored in the FNDR and INT broodlines of Yakima River spring Chinook salmon after one or two generations in culture. The INT broodline showed modest, but significant, differences in size at maturity, spawn date [41], body shape [57], fecundity [42], breeding success in an artificial channel [46, 58], vulnerability to predation [59] and spawning distributions [60] compared to the FNDR population. These early results suggested that phenotypic change resulting from hatchery culture may occur relatively rapidly even in a program designed to minimize such effects. More recently, evidence of rapid genetic divergence of the SEG broodline from the INT broodline of Yakima River spring Chinook salmon was observed by Waters et al. [16]. These investigators employed a genome-wide approach to document divergence over three generations of hatchery culture from BY 2001 to 2010. Divergence of the INT broodline from the FNDR population was minimal, but divergence of the SEG broodline was significant. They found evidence for a temporal trend in divergence at specific genomic regions, consistent with domestication selection [16]. Our results parallel that of Waters et al. [16] suggesting that the use of an integrated, as opposed to a segregated, breeding strategy has reduced the rate of change in the threshold sensitivity (PMRN) for early male maturation. Recall that the SEG broodline never experiences geneflow from precocious males, but the INT broodline may when spawning in the wild. Stated another way, this strategy has minimized divergence between the INT and FNDR broodlines relative to the SEG broodline and thus, helped maintain the wild phenotype.

Variation in PMRN midpoints in naturally rearing salmonid fishes has been attributed to both genetic effects as well as phenotypic plasticity. Piché et al. [38] modeled the threshold for early male maturation in different populations of naturally rearing Atlantic salmon, *Salmo salar*, in Nova Scotia, Canada. They used a common garden experimental construct with pure and mixed crosses and provided evidence of genetic variation in the size threshold for precocious parr maturation [38]. Alternatively, transplant studies in spotted charr, *Salvelinus leucomaenis* [61] demonstrated plasticity in the PMRN based on variation in fork length and environmental variation in river width and temperature throughout the Onbetsu River in Hokkaido, Japan. Specifically, increasing river width was associated with a lower PMRN while increasing length and higher stream temperature was associated with a higher PMRN [61]. Similarly, Baum et al. [62] demonstrated that spatial variation in growth rate as a response to riverine altitude affected the PMRN of Atlantic salmon in the River Spey, Scotland. Interestingly, they found that size-at-age was a good predictor of maturation rate, but across sites the relationship was influenced by altitude as well. However, it should be noted that, unlike the current investigation where fish were measured several months prior to maturation, in the work of Baum et al. [62] and Morita et al. [61] fish were collected near the time of spawning. Thus, their results may be more reflective of differences in growth opportunity long after the maturation decision has been made rather than differences in the PMRN.

Latitudinal variation in the distribution of salmonids has also been associated with variation in the PMRN [35]. In general, salmonid species at lower latitudes, most notably at the southern extreme of their distribution, have higher prevalence of resident life histories while species at higher latitudes tend to have higher prevalence of anadromous forms. Together, altitudinal and latitudinal variation in the prevalence of residency vs. anadromy and corresponding variation in the PMRN of naturally rearing stocks is hypothesized to be dictated, in part, by travel distance, water temperature and variation in marine and freshwater productivity and their corresponding effects on growth opportunity (reviewed in Dodsen et al. [35]). This intrinsic hydrogeographical variation in the PMRN of naturally rearing stocks may contribute to intrinsic differences in precocious male maturation rates of various hatchery stocks [29] and these factors may, in turn, be impacted by the extent of domestication selection they have experienced since being brought into culture as well as natural selection once they are released into the river and ocean.

Many studies of anthropogenic effects on PMRNs in salmonids have focused on the effects of selective commercial harvest on exploited fish stocks, often referred to as fisheries induced evolution (see Marshall and Browman [63] and references therein). To our knowledge only one other study has addressed the effect of hatchery culture on the PMRN of salmonids. Debes and Hutchings [64] examined the effects of zero, three and five generations of selection for rapid growth on precocious parr maturation in Atlantic salmon. They found size adjusted precocious male parr maturation probability was 34% in the wild strain, and reduced to 10% and 7% after three and five generations of selection; a reduction in the probability of maturation of 71% and 79%, respectively [64]. The strains of fish used in that investigation were from the Saint John River in New Brunswick, Canada and serve as the primary North American aquaculture strain and were specifically selected for high growth [65]. In contrast, the Yakima River spring Chinook salmon stock, studied in the current investigation, was a native wild stock that experienced essentially no hatchery influence prior to the programs implementation in 1997 and the program was specifically designed to minimize phenotypic and genotypic change to the stock [17]. Thus, seeing even a small, but significant, change in the age-2 minijack rate and the PMRN W_*P*50_ of the SEG broodline may represent an important change to the population since these fish were only two generations removed from being a wild stock.

Harstad et al. [29] monitored age-2 minijack rates at both segregated and integrated spring Chinook salmon hatchery programs throughout the Columbia River basin, USA and rates varied approximately 10-fold across programs ranging from 7.9% to 71.4%. A portion of the data from the INT broodline presented in Harstad et al. [29] is included in the current investigation. We found a significant positive relationship between size at smolt release and age-2 minijack rate in the integrated programs, but not the segregated programs surveyed and attributed this difference to domestication selection against early male maturation in hatchery culture. Precociously mature males are never used as broodstock in segregated hatchery programs. Furthermore, we found that the average age-2 minijack rate of the integrated hatchery programs was approximatey twice that of the segregated hatchery programs (42.1% vs. 21.8%) despite the fact that the segregated stocks were generally released at a larger average fork length [29]. Similarly, Spangenberg et al. [56] examined age-2 minijack rates and the PMRN in spring Chinook salmon from Carson National Fish Hatchery in WA, USA that has been segregated since the 1960s and the Hood River stock, Oregon, USA that has experienced variable levels of integration over the last two decades. We found lower age-2 minijack rates and a higher PMRN in the more segregated Carson stock compared to the more integrated Hood River stock when the two were reared under a common garden experimental design at Carson National Fish Hatchery [56]. The aformentioned studies provide evidence that, similar to wild salmonid populations [38], different hatchery strains possess different PMRNs. Furthermore, evidence from the current investigation suggests that modern supplementation hatchery breeding protocols that call for random mating among individuals, not neccessarily directed selection for fast growth like that described previously for aquaculture production [64, 65], may still result in significant shifts in this important life history trait. This suggests that integrated hatchery strategies may help to maintain some allelic contribution from precociously maturing males on the spawning grounds [66] and serve to maintain high age-2 minijack rates and a low PMRN in the INT broodline relative to the SEG broodline when these fish are brought into hatchery culture.

### Management implications

The consequences of hatchery induced shifts in early male maturation rates and the PMRN W_*P*50_ depend on the objective of the program. Segregated hatchery programs are designed to increase the number of anadromous adults available for harvest and broodstock relative to the number of smolts released (defined as the smolt-to-adult return rate or SAR). Previous studies in hatchery [67] and wild [68] salmonids have demonstrated that larger smolts survive at higher rates than smaller smolts within a location and release year cohort. Due to the shift toward a higher PMRN in well established segregated hatchery programs, one may be able to rear a larger smolt without incurring high rates of early male maturation, thus increasing the number of smolts, rather than precocious males, released and the number of adults returning (increase SARs) [22, 29]. Ironically, the segregated rearing strategy tends to reduce the number of minijacks produced, an outcome that is more in line with the 10-fold lower minijack rates estimated to be observed in wild relative to hatchery Yakima River spring Chinook salmon [22].

Integrated salmon hatchery programs are frequently designed to minimize genotypic and phenotypic changes to the supplemented population. As described in Harstad et al. [29], evidence suggests that wild stocks, naturally rearing stocks, and stocks with minimal hatchery influence appear to have relatively low PMRNs leading to the potential for very high rates of precocious male maturation when first introduced to hatchery culture conditions where they often experience optimal water temperatures and ample rations throughout the year. In the common garden portion of the current investigation (Part II), age-2 minijack rates were 68.3% and 58.6% in the INT (0–1) and SEG (2) broodlines, respectively. This significant decline in age-2 minijack rate observed in the SEG broodline after only two generations in culture suggests that traditional hatchery rearing practices, recognized for domestication [7], may result in rapid evolutionary change in the PMRN for precocious male maturation. It is important to recall that 100% of the broodstock in the Yakima Supplementation Program are natural origin (unmarked returning adults). This is a relatively unique situation among integrated hatchery programs and most do not reach this level of natural incorporation. So, it may be important to keep in mind that the degree of phenotypic change may be directly proportional to the degree of wild fish integration in a given program. Since most supplementation programs don’t achieve levels of wild integration approaching that of the Yakima Supplementation Program, the likelihood that these programs will experience a rapid increase in the PMRN W_P50_ for minijack maturation may be high.

While hatchery integration strategies that minimize generations in culture may slow the rate of change in genotype [16] and phenotypic characteristics like the PMRN W_*P*50_ compared to segregated programs, there is a paradoxical downside to this scenario. Integrated progams may maintain the potential to release significant numbers of males that will mature precociously [29]. Production of high numbers of precocious males means these fish are not available as potential full size anadromous adults. Previous studies have suggested that the survival following release of precociously mature hatchery spring Chinook salmon is relatively low [22, 25]. Those that do survive have been estimated to produce significantly less offspring during matings with naturally rearing anadromous females in studies of Yakima River [46] and Wenatchee River, WA spring Chinook salmon [66]. Furthermore, Ford et al. [6] also found a negative correlation between the relative reproductive success of spring Chinook salmon from the Wenatchee River supplementation program and that of their progeny spawning in the wild. They attributed the difference in reproductive success between the hatchery and naturally rearing fish to low relative reproductive success of hatchery age-2 minijacks spawning in the wild. But it should be noted that spawning site selection was also found to be a significant factor in the observed difference in relative reproductive success of wild and hatchery fish as well [69]. So, even a modest increase in precociously mature males may have a negative effect on supplemented populations.

High growth rates at specific times of the year in hatchery culture are associated with increased age-2 minijack rates, most notably in integrated stocks [29]. Early in juvenile development, age-2 minijacks represent males with high growth rate and a potentially low PMRN. Thus, selection against traits associated with early male maturation will increase the potential for domestication in these stocks. This potential for domestication is dependent on the size of the natural population being conserved and the proportion of hatchery origin spawners on the spawning grounds. This reality makes it imperative that hatchery supplementation programs designed to minimize genotypic and phenotypic changes to the native population be cognizant of the potentially low PMRN of these fish and design rearing programs to control growth and energy acquisition during specific sensitive physiological windows [43] to minimize potential genotypic and phenotypic changes to the supplemented stock.

Hatchery and Genetic Management Plans [18, 19] are technical documents used by resource and policy managers that describe the composition and operation of each salmon hatchery program in the Pacific Northwest United States of America. In the course of developing and implementing future Hatchery and Genetic Management Plans, managers may want to consider measuring age-2 minijack rates and the PMRN W _*P*50_ of supplemented stocks in order to track and model the potential for domestication in supplementation programs and to use this information to adjust rearing regimes accordingly.

## Supporting information

S1 Fig **Relationship between age-2 minijack rates and (A) unadjusted and (B) adjusted W**_***P*50**_ **estimates.** Progeny of SEG (black circles) and FNDR/INT (open grey squares) broodlines reared at Clark Flat acclimation site BYs 1998–2011. Adjusted PMRN W_*P*50_ estimates were adjusted for differences in winter growth between rearing groups.
(TIFF)

S2 Fig Relationship between age-2 minijack rates and estimated age-3 jack contribution to fertilized eggs. Progeny of SEG (black circles: *R*^*2*^ = 0.33, *N* = 9, *P* = 0.107), and FNDR/INT broodlines (open grey squares: *R*^*2*^ = 0.05, *N* = 10, *P* = 0.53) reared at Clark Flat acclimation site BYs 2002–2011. The outlier SEG BY 2006 was omitted from the SEG regression line.
(TIFF)

S1 Table Characterization of broodstock for each broodline and brood year. W = wild or natural origin (unmarked); H = hatchery origin (marked).
(XLSX)

S2 Table Mean monthly weight (WT, g) of juveniles reared at CESRF for each brood year. This data was collected and provided by CESRF staff. Specific growth rate (SGR) was calculated as: [(1nWT_2_/lnWT_1_)/(T_2_-T_1_)]*100 where *WT*_2_ = final weight (g) and *WT*_1_ = initial weight (g); time (*T*) was measured in days.
(XLSX)

S3 Table Logistic regression analysis of weight (*w*) broodline (*g*), and brood year (*y*) effects on age-2 minijack maturation, Part I. Results for full model with no interaction terms (Model 1) for individual male yearling spring Chinook salmon sampled at Clark Flat acclimation site in brood years 2002–2011. Model 2 also includes the interaction between broodline and brood year (*gy*). Broodline treatments codded as: SEG = 0; INT = 1 for statistical analysis. These results correspond with Fig 5A.
(XLSX)

S4 Table Logistic regression analysis of weight (*w*) and broodline (*g*) effects on age-2 minijack maturation by brood year, Part I. The categorical variable broodline was coded as: SEG = 0; INT or FNDR = 1. Only males were used in these analyses. The models from BYs 2002–2011 were used to predict the PMRN W_*P*50_ estimates for each broodline in each brood year that are presented in Figs 5 & 6.
(XLSX)

S5 Table Logistic regression analysis of weight (*w*) and broodline (*g*) effects on age-2 minijack maturation, Part II. This model was used to predict the reaction norms shown in Fig 10A and PMRN W_*P*50_ estimates in Fig 10B for each broodline treatment from the common garden experiment. Broodline treatments were coded as: SEG (2) = 0; SEG (1) = 1; INT (0–1) = 2 for statistical analysis. Only males were used in this analysis.
(XLSX)

S1 Text PMRN W _*P*50_ coding for STATA statistical software. (DOCX)
