# Supplementary figures and images for: Maintaining a wild phenotype in a conservation hatchery program for Chinook salmon: The effect of managed breeding on early male maturation
Source: PLoS One. 2019 May 15;14(5):e0216168. doi: 10.1371/journal.pone.0216168 (PMC6519831; doi:10.1371/journal.pone.0216168)

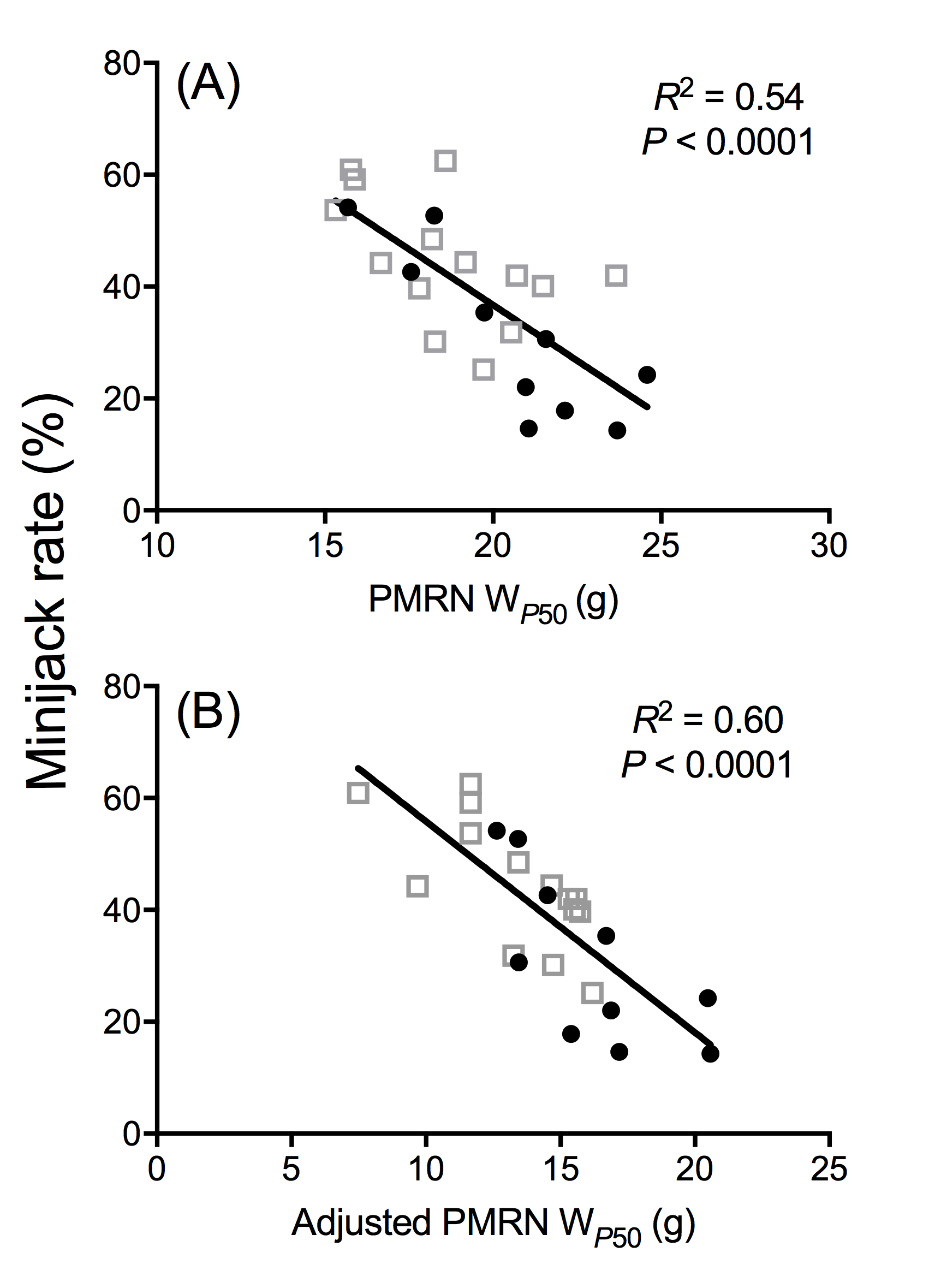

Supplement: S1 Fig — Relationship between age-2 minijack rates and (A) unadjusted and (B) adjusted WP50 estimates. Progeny of SEG (black circles) and FNDR/INT (open grey squares) broodlines reared at Clark Flat acclimation site BYs 1998–2011. Adjusted PMRN WP50 estimates were adjusted for differences in winter growth between rearing groups. (TIFF) [file pone.0216168.s001.tiff]

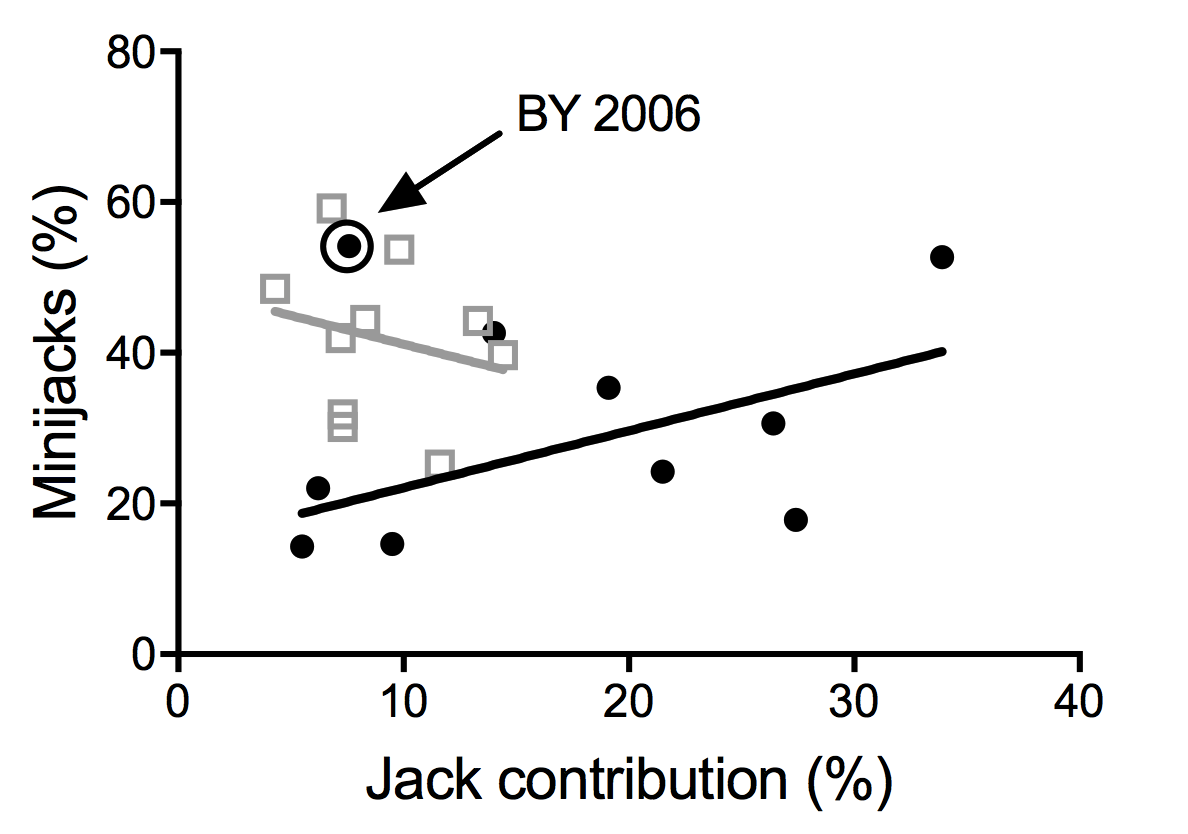

Supplement: S2 Fig — Progeny of SEG (black circles: R2 = 0.33, N = 9, P = 0.107), and FNDR/INT broodlines (open grey squares: R2 = 0.05, N = 10, P = 0.53) reared at Clark Flat acclimation site BYs 2002–2011. The outlier SEG BY 2006 was omitted from the SEG regression line. (TIFF) [file pone.0216168.s002.tiff]
