# Supplementary material for: Maintaining a wild phenotype in a conservation hatchery program for Chinook salmon: The effect of managed breeding on early male maturation
Source: PLoS One. 2019 May 15;14(5):e0216168. doi: 10.1371/journal.pone.0216168 (PMC6519831; doi:10.1371/journal.pone.0216168)
Supplement: S1 Text — (DOCX) [file pone.0216168.s008.docx]

**S1 Text.**

**Estimation of WP50 using STATA, Part I**

‘MAT’ = maturation status, binary variable (0 = immature, 1 = mature)

‘WT’ = weight (g), continuous variable

‘GENE’ = broodline, categorical variable (0 = SEG, 1 = INT/FNDR)

‘BY’ = brood year, categorical variable

Brood years with only one broodline (BYs 1998-2001):

. logit MAT WT

. nlcom-_b[_cons]/_b[WT]

Brood years with two broodlines (BYs 2002-2011; following example = BY 2002):

. logit MAT WT i.GENE if BY==2002

. nlcom -_b[_cons]/_b[WT] (for GENE category coded as ‘0’)

. nlcom (-(_b[_cons]+_b[1.GENE])/(_b[WT])) (for GENE category coded as ‘1’)

**Estimation of WP50 using STATA, Part II**

‘MAT’ = maturation status, binary variable (0 = immature, 1 = mature)

‘WT’ = weight (g), continuous variable

‘GENE’ = broodline, categorical variable (0 = SEG 2, 1 = SEG 1, 2 = INT 0-1)

. logit MAT WT i.GENE

. nlcom -_b[_cons]/_b[AWT] (for GENE category coded as ‘0’)

. nlcom (-(_b[_cons]+_b[1.GENE])/(_b[WT])) (for GENE category coded as ‘1’)

. nlcom (-(_b[_cons]+_b[2.GENE])/(_b[WT])) (for GENE category coded as ‘2’)
